# Multiscale entropy of ADHD children during resting state condition

**DOI:** 10.1007/s11571-022-09869-0

**Published:** 2022-08-30

**Authors:** Brenda Y. Angulo-Ruiz, Vanesa Muñoz, Elena I. Rodríguez-Martínez, Celia Cabello-Navarro, Carlos M. Gómez

**Affiliations:** Human Psychobiology Laboratory, Experimental Psychology Department, University of Seville, C/Camilo José Cela S/N, 41018 Seville, Spain

**Keywords:** ADHD, Complexity metrics, Multiscale entropy, Power spectral density, Variability, Resting-state

## Abstract

This present study aims to investigate neural mechanisms underlying ADHD compared to healthy children through the analysis of the complexity and the variability of the EEG brain signal using multiscale entropy (MSE), EEG signal standard deviation (SDs), as well as the mean, standard deviation (SDp) and coefficient of variation (CV) of absolute spectral power (PSD). For this purpose, a sample of children diagnosed with attention-deficit/hyperactivity disorder (ADHD) between 6 and 17 years old were selected based on the number of trials and diagnostic agreement, 32 for the open-eyes (OE) experimental condition and 25 children for the close-eyes (CE) experimental condition. Healthy control subjects were age- and gender-matched with the ADHD group. The MSE and SDs of resting-state EEG activity were calculated on 34 time scales using a coarse-grained procedure. In addition, the PSD was averaged in delta, theta, alpha, and beta frequency bands, and its mean, SDp, and CV were calculated. The results show that the MSE changes with age during development, increases as the number of scales increases and has a higher amplitude in controls than in ADHD. The absolute PSD results show CV differences between subjects in low and beta frequency bands, with higher variability values in the ADHD group. All these results suggest an increased EEG variability and reduced complexity in ADHD compared to controls.

**Supplementary Information:**

The online version contains supplementary material available at 10.1007/s11571-022-09869-0.

## Introduction

One of the neurodevelopmental disorders commonly diagnosed during childhood is Attention Deficit Hyperactivity Disorder (ADHD). Its main symptoms include attention deficit, hyperactivity, and/or impulsivity. The disorder affects the normal development, curricular activities, and performance of the child, leading to a high risk of learning and social problems (Magnin & Maurs 2017). The neuropsychological differences observed in these children are being explained through two neurocognitive models: the *maturational delay model*, and the *differential development model*. The *maturational delay model* suggests a protracted development of children with ADHD compared to normal children (i.e., ADHD children would present both EEG and behavioral parameters of younger control children; Kinsbourne 1973; Matsuura et al. 1993). The *differential development model* suggests a different pattern of parameters on the developmental trajectory is observed in ADHD subjects, when compared to controls, due to the genetic or biographical background (Giertuga et al. 2017; Saad et al. 2018; Rodríguez-Martínez et al. 2020).

Several studies have analyzed the neural correlates of ADHD, especially those related to quantitative measures of the brain signal through EEG (Newson & Thiagarajan 2019; Clarke et al. 2020). These have been mainly based on linear measures, such as those involving spectral power analysis, which has shown an atypical neuronal pattern in resting-state conditions (Clarke et al. 2020) and during cognitive information processing (Nazari et al. 2011). Spectral power reductions in fast frequency bands (beta) increases in slow frequency bands (delta and theta; Barry et al. 2009; Newson & Thiagarajan 2019; Clarke et al. 2020; Rodríguez-Martínez et al. 2020). Moreover, the theta/beta ratio (TBR) and the theta/alpha have been reported as possible ADHD differential markers (Barry et al. 2003, 2009). Nevertheless, the results are highly variable. For instance, Giertuga et al. (2017) showed a decrease in all frequency bands in ADHD compared to controls. There are still uncertainties regarding the use of the TBR for diagnosis (Arns et al. 2013; Saad et al. 2018) leading to a need for precise measures that would allow more accurate characterization of ADHD neurophysiological differences compared to control.

In the last decades, nonlinear analyses of EEG signals, such as the variability and dynamic complexity of the underlying neural networks have been implemented (Takahashi et al. 2009, 2013; Garret et al. 2013; Van Noordt & Willoughby 2021). These are based on the idea that the human brain is a complex nonlinear system, and its complex spatial and temporal fluctuations are part of its intrinsic activity (Takahashi et al. 2009). In this sense, nonlinear analyses could detect subtle changes and therefore new methods of interpreting complex neural dynamics (Pincus 2006; Sohn et al. 2010). Variability provides an estimate of the range of values of the neural signals and can be measured through different measures such as the standard deviation (SD) (Garret et al. 2013) or the coefficient of variation (CV) (Angulo-Ruiz, et al. 2021). Complexity, on the other hand, quantifies the information included in these signals and examines the irregularity or predictability across one, or, multiple time scales (Garret et al. 2013). Therefore, it determines the probability of finding specific or similar patterns repeating in a time series (Costa et al. 2002, 2005). It is measured through techniques such as entropy (Kolmogorov 1958; Pincus 1991, 1995; Richman & Moorman 2000; Takahashi 2013), or multiscale entropy (MSE) (Costa et al. 2002, 2005; Takahashi 2013). MSE implies intrinsic physiological complexity, allowing the differentiation between noise and meaningful complexity, and detecting long-range temporal correlations, due to the analysis across multiple temporal scales (Costa et al. 2002, 2005; Takahashi et al. 2009). Thus, MSE can, first, reflect neurophysiological dynamics, and second, indicate an atypical pattern implying a brain disease condition (Costa et al. 2002; Mizuno et al. 2010). A recent study by Bosl et al. (2022) found that the scales (from fine to coarse) measured in the coarse-graining process are related to the frequency bands of the power spectral density (PSD), establishing a direct relationship between these two metrics.

Studies related to these measures have pointed out that variability and complexity increase during development (McIntosh et al. 2008; Lippe et al. 2009; Garret et al. 2013; Szostakiwskyj et al. 2017; Van Noorddt & Willoughby 2021; Angulo-Ruiz et al. 2021), and decrease with normal aging (Takahashi et al. 2009; Nomi et al. 2017). However, regarding variability, a possible regional specificity (Nomi et al. 2017) and the oscillatory frequencies (Angulo-Ruiz et al. 2021) must be taken into account as potential factors influencing the study of EEG maturation. Moreover, complexity has shown more significant increases and changes in fronto-central regions (Van Noordt & Willoughby 2021) during maturation. Szostakiwskyj et al. (2017) found an increase in MSE with age for fine scales, and a MSE decrease for coarse scales. These terms are identifiers of the range of time scales calculated in MSE. Thus, the fine scales correspond to a situation in which few signal points (individual time samples) are averaged, and the coarse scales when many signal points are averaged. It is important to note that currently there is no standard range for the differentiation of the scales (Shen et al. 2021).

Complexity has been studied among various clinical populations with mental (Takahashi et al. 2010; Fernández et al. 2013; Li et al. 2018) and neurodevelopmental disorders (Catarino et al. 2011; Bosl et al. 2011, 2017; Chu et al. 2017). In ADHD the results are still few, inconsistent and heterogeneous, showing diverse or even contradictory results (Fernández et al. 2009; Sohn et al. 2010; Gómez et al. 2013; Sokunbi et al. 2013; Li et al. 2016; Rezaeezadeh et al. 2020; Hu et al. 2021). Specifically, in EEG, increased complexity (Li et al. 2016) and decreased complexity (Rezaeezadeh et al. 2020) has been reported in children with ADHD. Table 1 shows a review of the differences in complexity metrics of ADHD and controls. In general, there is an agreement of an abnormal EEG complexity for brain disorders (Takahashi 2013; Chu et al. 2017). Thus, an increase or decrease in complexity could result in inefficient information processing (Ghosh et al. 2008; McIntosh et al. 2008, 2010), abnormal underlying physiological dynamics (Takahashi 2013), and aberrant neuronal connectivity (Takahashi et al. 2016). Therefore, it is suggested that healthy brains are more variable and complex (Garrett et al. 2013), and abnormal levels of variability and/or complexity, may be related to suboptimal cognition (Nomi et al. 2018; Easson & McIntosh 2019).

**Table 1:** Review of complexity articles in ADHD and control groups

Article	Technique	Measure	Experimental Condition	Control	ADHD	Results
Rezaeezadeh et al. (2020)	EEG	MSE, and others	Resting State (CE)	12	12	ADHD has a more regular neural system. Reduced dynamic complexity
Papaioannou et al. (2021)	EEG	MSE	Tasks	24 adults	30 adults	ADHD: MSE is higher in ADHD than in controls during the task
Li et al. (2016)	EEG	MSE	Tasks	13	13	ADHD: Higher complexity in delta and theta frequency bands; lower complexity in alpha. Aberrant neural connectivity
Hu et al. (2021)	fNIRS	MSE	Resting State	41	42	ADHD reduced brain signal variability in higher order primary brain networks (DMN, frontoparietal attention, and visual networks)
Sohn et al. (2010)	EEG	ApEn	Resting State (OE)	12	11	ADHD: Lower ApEn in right frontal regions at the task, but not at rest
Sokunbi et al. (2013)	fMRI	SampEn	Resting State	13 adults	17 adults	ADHD: Less complexity. Reduced entropy in frontal and occipital regions bilaterally. Significant negative correlation between symptoms and entropy
Gómez et al. (2013)	MEG	FuzzyEn	Resting State (CE)	14	14	ADHD more regular than controls
Fernández et al. (2009)	MEG	LZV	Resting state (CE)	14	14	Higher LZV in control than in ADHD

*EEG*: Electroencephalogram; *fNIRS*: Functional Near-Infrared Spectroscopy; *MEG*: Magnetoencephalography; *fMRI*: Functional Magnetic Resonance Imaging; *ADHD*: Attention Deficit Hyperactivity Disorder; *CE*: Closed Eyes; *OE*: Open Eyes; *MSE*: Multiscale Entropy; *ApEn*: Approximate Entropy; *SampEn*: Sample Entropy; *FuzzyEn*: Fuzzy Entropy; *LZV*: Lempen-Ziv Complexity; *DMN*: Default Mode Network

We propose to analyze the brain signal complexity of EEG using MSE to explore the underlying neural mechanisms of ADHD children compared to a sample of healthy children. MSE was computed in Open Eyes (OE) and Closed Eyes (CE) experimental conditions given that MSE is a sensitive metric to characterize nonlinear abnormalities in brain diseases (Chu et al. 2017; Shen et al. 2021) in resting-state conditions. However, no such application has been approved for clinical use yet. Additionally, the analysis of the SDs EEG (Standard Deviation of the EEG at the different scales), absolute PSD in different brain rhythms (mean, standard deviation across trials (SDp), coefficient of variation across trials (CV)), and mean of relative PSD were computed, as complementary measurements to raise a comprehensive approach to characterize controls and ADHD. The present study hypothesizes that (i) children with ADHD will manifest an atypical neural pattern of complexity compared to normo-developmental children. In addition, although most studies have shown an increase in MSE with age for all scales analyzed, Szostakiwskyj et al., (2017) have shown positive and negative slopes depending on the scales for the relationship between MSE and age, so we intend to test the constancy or variability of these slopes in both groups. (ii) For SDs, we expect a decrease in EEG variability with age in both groups for all the scales, associated with the reduction in EEG amplitude with age. Comparison between groups would indicate whether EEG variability may also be a suitable metric to differentiate ADHD from controls; (iii) for absolute PSD, and SDp we expect a decrease with age, and amplitude differences between groups for low-frequency bands, as well as, variability differences with age when normalizing by the PSD mean of different brain rhythms (CV), and (iv) an inverse relationship with age of low frequency bands of relative PSD, while a positive relationship for high frequencies, taking into account that in MSE higher scales represent increasingly lower frequency bands, with all scales containing the lowest frequencies (Bosl et al. 2022). This approach intends to provide findings to discriminate between neurophysiological metrics based on EEG variability, complexity, and power in controls and ADHD subjects, to explain the possible neural causes of the disorder, and in turn to find biological markers that would facilitate diagnosis.

## Methods

### Participants

A group of children and adolescents diagnosed with ADHD by clinical experts from two public hospital services participated in this study. A structured interview and the DuPaul parent questionnaire (DuPaul et al. 1998) were conducted for the diagnosis. The ADHD group, consisting of 40 children and adolescents aged 6 to 17 years, was recorded under experimental OE and CE conditions. Only subjects who (i) obtained a minimum of 50 trials without artifacts in one or both conditions (OE and CE), and (ii) showed diagnostic agreement between the administered questionnaire and the clinical diagnosis were selected for data analysis. Therefore, the ADHD group was finally composed of 32 children and adolescents for the OE condition (M = 10.94, SD = 3.18, 25 males, 7 females, 6–17 years) and 25 ADHD children and adolescents for the CE condition (M = 11.8, SD = 3, 21 males, 4 females, 7–17 years). Grouping by ADHD typology was not performed due to the low number of subjects in each group.

Thirty-two control subjects, matched in age and gender to ADHD in the experimental condition for OE (M = 10.84, SD = 3.10, 25 males, 7 females, 6–17 years) and CE (M = 11.68, SD = 2.93, 21 males, 4 females, 7–17 years), were selected from public schools through accessibility sampling. The control subjects for CE were reduced to 25 to equate them to the number of ADHD subjects in the CE condition. There were no significant differences between the two groups in age (OE: (F (1, 62) = 0.000042, p = 0.995; eta partial squared = 0.0000007) and CE: (F (1, 48) = 0.000122, p = 0.991, eta partial squared = 0.000003) or gender (OE: (F (1, 62) = 0.00, p = 1; eta partial squared = 0.00) and CE: (F (1, 48) = 0.00, p = 0.991, eta partial squared = 0.00). The equating in gender and age between controls and ADHD allowed to eliminate these factors for mean comparisons statistical analyses.

Controls did not report neurological diseases, signs of epileptic discharges, or psychological impairments. The experimental protocol was approved by the biomedical research ethics committee of the autonomous community of Andalucía. The guidelines of the Declaration of Helsinki were followed and written informed consents were obtained from the parents.

### Experimental session

Spontaneous EEG activity was obtained in the OE and CE experimental conditions with a duration of 3 min. Subjects were instructed to stay still and to maintain a state of relaxation in both experimental conditions. In the OE condition, they were also instructed to blink as little as possible and to look at a cross in the center of the screen.

A 32-electrode cap (ELECTROCAP) (Fp1, Fpz, Fp2, F7, F3, Fz, F4, F8, FC5, FC1, FC2, FC6, M1, T7, C3, Cz, C4, T8, M2, CP5, CP1, CP2, CP6, P7, P3, Pz, P4, P8, POz, O1, Oz, O2) assembled according to the international 10–20 system was used for recording. Electrodes placed on the scalp were referenced off-line to the mean mastoid (M1 + M2)/2. Horizontal eye movements were recorded with two electrodes placed on the outer edge of each eye and vertical movements with two electrodes placed above and below the left eye. Impedance was obtained below 10 Kohms. Using an analog-to-digital acquisition and analysis system (ANT amplifiers, The Netherlands), data were recorded with a gain of 20,000 in direct current at 512 Hz without any filtering.

### Data analysis

For EEG data analyses, the EEGLAB toolboxes (Delorme & Makeig 2004) and Matlab R2019a software package were employed.

The EEG signal was band-pass filtered from 0.5 to 35 Hz (*eegfiltnew* EEGLAB function). The Artifact Subspace Reconstruction (ASR) algorithm was applied to correct EEG signal artifacts that exceeded 20 times the standard deviation of the calibrated data (*clean rawdata* EEGLAB function). Data were reconstructed and epochs (2 s in duration) that exceeded ± 120 μV in any channel were rejected for subsequent analysis (*eegthresh* EEGLAB function). Subjects with less than 50 trials were not further analyzed. Table 2 shows the number of epochs accepted in each group and experimental condition. ANOVA comparisons showed no differences in the number of epochs accepted in both groups: For the OE condition (F (1, 62) = 0.147, *p* = 0.703, eta partial square = 0.002) and for the CE condition (F (1, 48) = 1.3, p = 0.259, eta partial square = 0.026).

**Table 2:** Mean and Standard Deviation of accepted trials in controls and ADHD subjects in open (OE) and closed eyes (CE) conditions

		Control		ADHD	
		OE	CE	OE	CE
Trials	Mean	82.56	84.84	81.69	81.96
	SD	8.67	5.36	9.6	11.42

### Multiscale entropy analysis

MSE was computed for all channels (except M1 and M2) with the "*multiscaleSampleEntropy*" function of Matlab (Malik 2022) based on Costa et al. (2005). MSE analysis is a derivation of Shannon entropy (Shannon & Weaver 1949) and Pincus approximate entropy (Pincus 1991). It is based on the calculation of the sampling entropy (SampEn) of the EEG signal at multiple time scales (Costa et al. 2002, 2005; Richman & Moorman 2000). MSE is an index of signal complexity (Garrett et al. 2013) and is computed using a process known as coarse-graining. Each time scale is defined by averaging the different neighboring points of the original time series (of length τ), dividing the EEG signal in non-overlapping windows of a different number of samples. Subsequently, the SampEn is calculated for each time scale. This analysis evaluates the similarity of the repetition frequency of patterns of m data points (*p*^m) versus another of m + 1 points (*p*^(m + 1)). It is necessary, for this purpose, to define a similarity limit (r) that delimits the tolerance range for individual data points to be considered similar (k). The similarity limit is normalized by the EEG Standard Deviation (SD) k < r × SD (Malik 2022).

Recently, it has been suggested that the coarse-grained process is comparable to Haar wavelet approximations on power-of-two scales, relating the different frequency bands of traditional spectral power analysis to different scales of the MSE (Bosl et al. 2022). In this sense, the coarser scales would contain the lower frequencies with filtering of the high frequencies (Kosciessa et al. 2020), and the lower scales are the original signal with the high frequencies, with all scales containing also low frequencies (Bosl et al. 2022).

In our study, we set the parameters m = 2 and r = 0.5 considering the recommendations given by previous EEG signal complexity studies (Richman & Moorman 2000; McIntosh et al. 2008; Miskovic et al. 2016; Kosciessa et al. 2020; Kloosterman et al. 2019), in which the SD parameter permits normalizing the r parameter by the EEG standard deviation in each particular scale. MSE was calculated for time scale 1 to 34, which allows analysis of entropy from the finest to the coarsest scales, as well as indirect analysis of low frequency bands (≤ 7.73 Hz). The last time scale corresponds to a time of 64.6 ms per time point and 31 time points per trial.

The equation used to compute SampEn was (Malik 2022), following the notation of Kosciessa et al. (2020):

$$\mathrm{SampEn}=log\frac{ {p}^{m}(r)}{{p}^{(m+1)}(r)}$$

High values of SampEn would indicate the presence of low temporal regularity or high complexity (i.e., many patterns of length m do not repeat over length m + 1) whereas low values would indicate high similarity/regularity or low complexity indicating the poverty of information (McIntosh et al. 2008; Garrett et al. 2013).

### EEG standard deviation analysis

The EEG standard deviation (SDs) was computed in the same scales as MSE for both conditions and groups. The EEG SDs were computed as the EEG variability in each trial, and then, the mean of SDs across trials was obtained in the different scales. This parameter could inform if the variability of the EEG was different between both groups at the different scales and at different ages (by correlating SDs with age). The SDs parameter provides the basal variability of EEG and would be complementary to the PSD, which provides the energy of each EEG frequency, and the MSE, which provides the data complexity.

### Absolute and relative PSD analyses

The mean of absolute PSD in each trial was calculated for each subject in both experimental conditions (OE and CE). PSD was calculated in 2 s windows (1024 sampling points at a sampling rate of 512 Hz) with the EEGLAB *spectopo* function which employs the Matlab *pwelch* function applying a hamming window. As *spectopo* calculates the logarithm of absolute PSD (Y = 10 * Log (PSD)), each subject's PSD values (PSD mean (M) and standard deviation (SDp) across trials) were calculated by removing the logarithms (PSD = e^Y/10^) to visualize the data and calculate the coefficient of variation (CV) across trials. Four frequency bands averaged in different ranges were taken into account for subsequent analysis: delta (1–2 Hz), theta (4–7 Hz), alpha (8–11 Hz), and beta (13–20 Hz). The gamma band was not calculated due to the bandpass filter (0.5–35 Hz) used in the data analysis in order to eliminate high-frequency electromyographical artifacts.

Relative PSD was calculated using the mean of absolute PSD (removing logarithms) of each subject at each electrode and using the following formula:

$${X}_{(fi)}=\frac{{PSD}_{(fi)}}{{\sum }_{i=1}^{20}{PSD}_{(fi)}}*100$$

where *X*(fi) is the relative PSD for a given frequency, PSD (*fi)* is the absolute PSD for a given frequency, and $\sum PSD(fi)$ is the sum of absolute PSD across all the considered frequencies (1–20 Hz). This analysis was performed for controls and ADHD in both experimental conditions (OE and CE).

### Statistical analysis

**Fig. 1:**
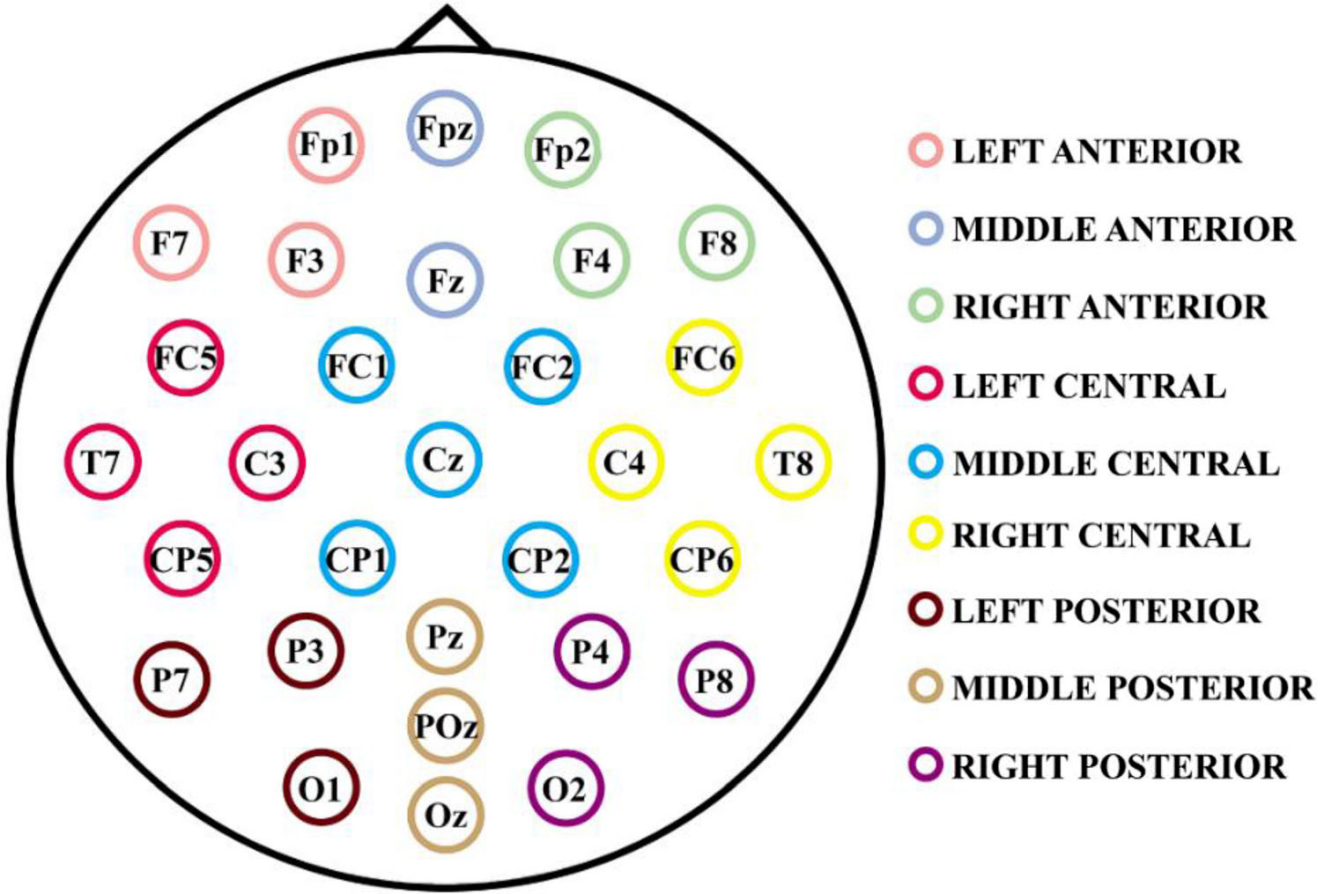
Localization and collapse of electrodes by regions. The colors indicate the nine defined scalp areas for electrodes collapse. The 30 electrodes are divided into two spatial dimensions (lateral and anterior–posterior) which have three values each one: left, middle, and right; and anterior, central, and posterior, respectively

To calculate the difference between groups of MSE, SDs, absolute PSD (mean and CV), and mean of relative PSD metrics, the values of these parameters in neighboring electrodes, as defined in Fig. 1, were collapsed to reduce the dimensionality of the data (Fig. 1), for both OE and CE. For the same purpose, the MSE results for the 34 temporal scales were organized into three broader scales (values in ms correspond to the scales time sampling): fine scales (1.9 ms (scale 1)—24.7 ms (scale 13)); medium scales (26.6 ms (scale 14)—43.7 ms (scale 23)); and coarse scales (45.6 ms (scale 24)—64.6 ms (scale 34)) as proposed by Szostakiwskyj et al. (2017). The period of the scales was computed by multiplying the EEG sample period by the scale order.

Employing the Statistical Package for the Social Sciences 25 (SPSS) three analyses of variance (ANOVA) were performed with the mean of the MSE for each type of scale (fine, medium, and coarse) in each brain area as defined above and in Fig. 1. For the first and second ANOVA, the within-subject factors were: type of scale (levels: fine, medium, and coarse), anteroposterior areas (levels: anterior, central, and posterior), and lateral areas (levels: left, medial and right); and the between-subject factor was the group (Control and ADHD subjects). The first two ANOVAs correspond to the independent analysis of OE and CE conditions, respectively. And for the third ANOVA, the experimental condition (OE and CE) was added as a within-subject factor, maintaining the previously considered factors. To have the same subjects in both OE and CE conditions only 23 controls and 25 ADHD remained for the third ANOVA, respectively. When significant group differences were found in the ANOVA analysis, the independent samples t-test (False Discovery Rate (FDR) corrected) were computed as post-hoc tests (Benjamini & Hochberg 1995). Statistically significant results of ANOVA are presented for all the considered factors, but only those including the group factor were discussed and analyzed in post-hoc tests, given that the main objective of the present report is the between-groups differences.

Additionally, a Spearman correlation analysis of the MSE with the age of the subjects (expressed in days) was applied to controls and ADHD independently, in both experimental conditions (OE and CE). For the correlational analysis, the values of MSE in all the electrodes were collapsed to reduce dimensionality. The collapse of electrodes was also applied for other variables in which correlational analysis was computed.

The SDs of the EEG for the 34 scales, collapsed by electrodes, followed the same statistical analysis procedure as the MSE. (i) Spearman correlation with age, (ii) Three analyses of variance (ANOVA), with and without factor "experimental condition" (OE and CE), and (iii) Independent samples t-tests (FDR corrected) for post-hoc analysis.

For absolute PSD statistical analysis, Spearman correlation was performed between the collapse of the mean PSD across electrodes for each considered frequency (1–20 Hz) and the age of the subject (expressed in days). The same correlational analysis was calculated for SDp, CV, and relative PSD. Additionally, for the relative PSD, a correlation analysis was performed between the relative PSD at different frequencies with the different MSE scales for each group and experimental condition. The coefficient of variation was calculated by dividing the standard deviation across trials of absolute PSD (SDp) by the mean of the absolute PSD (CV = SDp/M) in all subjects and the two experimental conditions (OE and CE) and each frequency (1–20 Hz). The means of absolute and relative PSD and CV were analyzed through three ANOVAs. The first (for OE condition), and second (for CE condition) ANOVA included as within-subjects factors: anteroposterior areas, and lateral areas, and as a between-subject factor the group. The third ANOVA added the within-subject factor "experimental condition" (OE vs CE). The ANOVAs were computed independently for the four different considered frequency bands (delta, theta, alpha, and beta).

The FDR, which was applied to the post-hoc and to correlational analysis, was calculated according to Benjamini & Hochberg (1995) as a control measure for multiple comparisons and Spearman correlations.

## Results

**Fig. 2:**
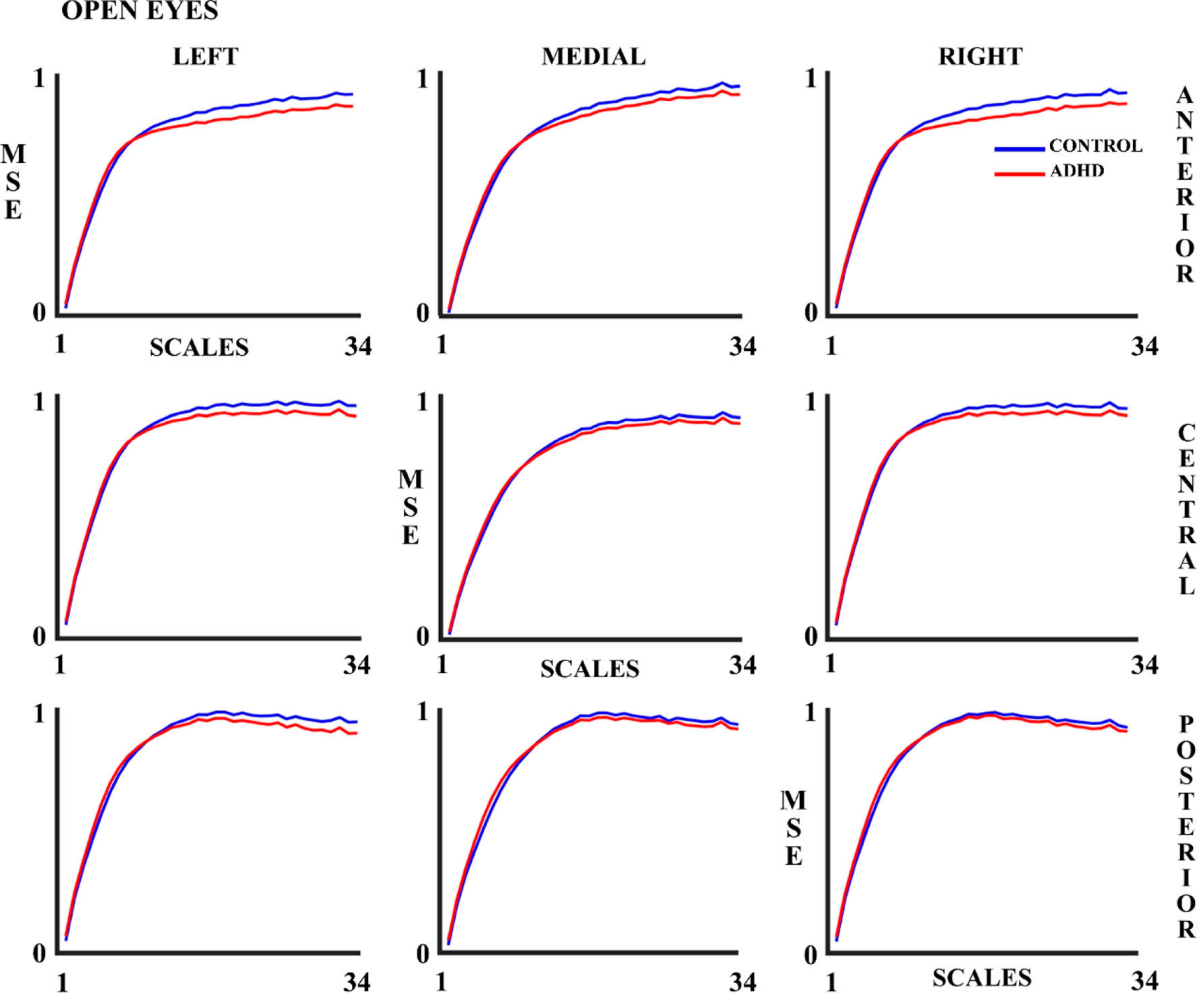
Multiscale Entropy (MSE) for 34 scales in control and ADHD subjects in the open eyes (OE) experimental condition in all the 9 considered areas. The blue line represents the control group and the red line the ADHD group

**Fig. 3:**
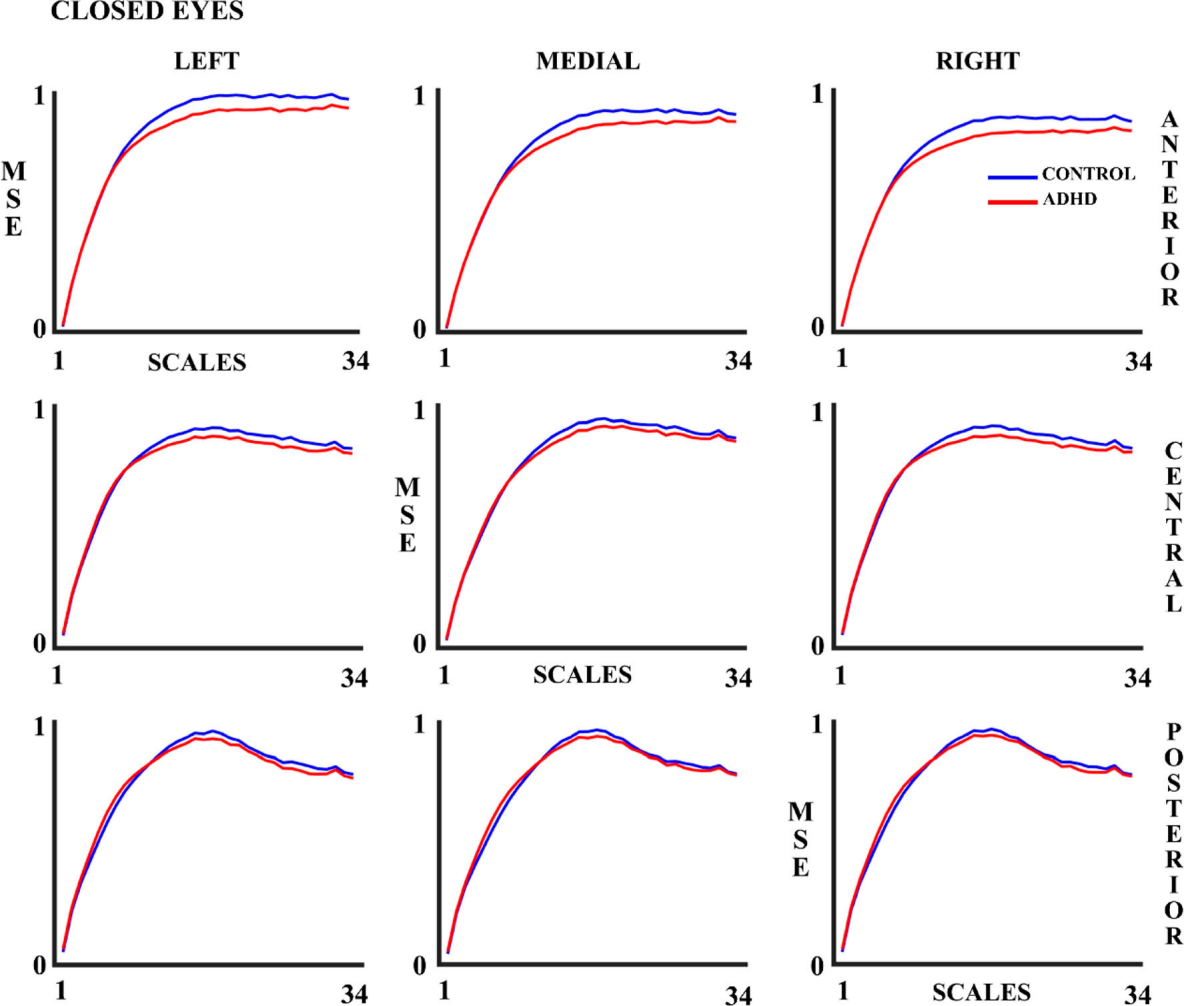
Multiscale Entropy (MSE) for 34 scales in control and ADHD subjects in the closed eyes (CE) experimental condition in all the 9 considered areas. The blue line represents the control group and the red line the ADHD group

Figures 2 and 3 show the MSE results in the OE and CE experimental conditions, respectively. The MSE increases as the number of scales increases. Although the figures suggest that the ADHD group presents lower MSE values compared to the control group in both OE and CE, the ANOVA (Table 3) shows that the statistically significant differences between Control and ADHD (MSE controls > MSE ADHD) groups were only present in the OE condition. As indicated in the methods section, an ANOVA including the experimental condition (OE & CE) as a within-group factor was performed for subjects presenting enough number of trials (Table 4). The latter ANOVA showed statistically significant differences between the control and ADHD groups.

**Table 3:** Significant results of the ANOVA analysis of the Multiscale Entropy values, with factors group of subjects (control and ADHD), scales (fine, medium, and coarse), laterality, and anterior–posterior, for open eyes (OE) and closed eyes (CE) conditions, independently

Open eyes	Between-subjects: *Group p* = .046* F = 4.14, gl = [1, 62], eta partial squared = .063 Within-subjects: Scales *p* < .001 F = 485.97, gl = [1.24, 76.66], eta partial squared = .887 Anterior–posterior *p* < .001 F = 114.83, gl = [1.22, 75.74], eta partial squared = .649 Scales x Laterality *p* < .001 F = 237.97, gl = [2.39, 148.28], eta partial squared = .793 Scales x Anterior–posterior *p* < .001 F = 45.04, gl = [2.03, 125.58], eta partial squared = .421 Laterality x Anterior–posterior *p* < .001 F = 14.07, gl = [3.28, 203.39], eta partial squared = .185 Scales x Laterality x Anterior–posterior *p* < .001 F = 45.88, gl = [4.61, 285.96], eta partial squared = .425
Closed eyes	Within-subjects: Scales *p* < .001 F = 383.11, gl = [1.46, 70.15], eta partial squared = .889 Laterality *p* < .001 F = 17.73, gl = [1.97, 94.47], eta partial squared = .270 Anterior–posterior *p* < .001 F = 120.59, gl = [1.3, 61.24], eta partial squared = .715 Scales x Laterality *p* < .001 F = 129.35, gl = [2.5, 120.51], eta partial squared = .729 Scales x Anterior–posterior *p* < .001 F = 107.14, gl = [2.59, 124.26], eta partial squared = .621 Laterality x Anterior–posterior *p* < .001 F = 10.42, gl = [2.64, 126.73], eta partial squared = .178 Scales x Laterality x Anterior–posterior *p* < .001 F = 35.89, gl = [4.19, 201.44], eta partial squared = .428

All significant results are displayed. The results in which the group factor was significant as a main or interactive effect are indicated with an asterisk. Please notice that gender and age were equated for control and ADHD groups

**Table 4:** Significant results of the ANOVA analysis of the Multiscale Entropy values, with factors group of subjects (control and ADHD), scales (fine, medium, and coarse), laterality, anterior–posterior, and open eyes and closed eyes conditions (OE & CE)

Between-subjects	Group *p* = .025* F = 5.33, gl = [1, 46], eta partial squared = .104
Within-subjects	Scales *p* < .001 F = 381.8, gl = [1.33, 61.38], eta partial squared = .892 Laterality *p* < .001 F = 10.05, gl = [1.96, 90.59], eta partial squared = .179 Anterior–posterior *p* < .001 F = 113.20, gl = [1.25, 57.61], eta partial squared = .711 OE-CE x Scales *p* < .001 F = 23.08, gl = [1.46, 67.23], eta partial squared = .334 OE-CE x Laterality *p* = .005 F = 5.75, gl = [1.93, 88.93], eta partial squared = .111 Scales x Laterality *p* < .001 F = 184.59, gl = [2.4, 110.52], eta partial squared = .801 OE-CE x Scales x Laterality *p* < .001 F = 22.37, gl = [2.63, 121.27], eta partial squared = .327 OE-CE x Anterior–posterior *p* < .001 F = 18.19, gl = [1.42, 65.51], eta partial squared = .283 Scales x Anterior–posterior *p* < .001 F = 84.42, gl = [2.3, 105.92], eta partial squared = .647 OE-CE x Scales x Anterior–posterior *p* < .001 F = 27.04, gl = [2.6, 118.42], eta partial squared = .370 Laterality x Anterior–posterior *p* < .001 F = 12.28, gl = [2.88, 132.57], eta partial squared = .211 Scales x Laterality x Anterior–posterior *p* < .001 F = 51.19, gl = [3.99, 183.64], eta partial squared = .527 OE-CE x Scales x Laterality x Anterior–posterior *p* = .015 F = 2.9, gl = [5.02, 230.75], eta partial squared = .059

All significant results are displayed. The results in which the group factor was significant as a main or interactive effect, are indicated with an asterisk. Please notice that gender and age were equated for control and ADHD groups

While Spearman correlations (Tables 5 and 6) show a positive correlation of the MSE with the age of the subjects (in days) for fine scales, this relationship reverses in coarser scales, and such for both experimental conditions (OE and CE) and for both groups (control and ADHD). A greater correlation (in absolute values) was observed for controls (M = 0.521, SD = 0.172) when compared to ADHD subjects (M = 0.356, SD = 0.156) in the CE condition (p < 0.001), (based on a t-test between the absolute correlation values).

**Table 5:** MSE at different scales vs. age (expressed in days) Spearman correlations (Rho) for control and ADHD group in Open Eyes (OE) condition

Scales	Control		ADHD	
	Rho	*p*	Rho	*p*
1	.534	.004	.411	.037
2	.551	.004	.431	.066
3	.541	.004	.424	.044
4	.500	.008	.407	.035
5	.491	.009	.404	.035
6	.490	.009	.409	.036
7	.545	.004	.418	.035
8	.607	< .001	.422	.035
9	.700	< .001	.427	.063
10	.729	< .001	.401	.035
11	.739	< .001	.396	.035
12	.761	< .001	.335	.079
**13**	**.739**	**< .001**	**.312**	**.103**
14	.722	< .001	.225	.253
15	.655	< .001	.109	.586
16	.593	< .001	− .019	.652
17	.488	.009	− .088	.917
18	.427	.026	− .128	.531
19	.351	.075	− .209	.284
20	.242	.247	− .298	.119
21	.196	.332	− .375	.046
22	.128	.516	− .426	.051
**23**	**.027**	**.883**	**− .424**	**.041**
24	− .074	.71	− .398	.036
25	− .146	.466	− .434	.088
26	− .169	.40	− .423	.038
27	− .200	.331	− .426	.056
28	− .203	.334	− .446	.089
29	− .223	.289	− .433	.076
30	− .269	.194	− .467	.012
31	− .376	.054	− .422	.036
32	− .306	.130	− .452	.011
33	− .422	.027	− .425	.047
34	− .478	.011	− .484	.017

*P-values* with FDR corrections for multiple comparisons. The MSE values for all the electrodes were collapsed to compute the correlations. The limit of the scales is indicated by bold. Notice the transition from positive to negative correlations as scale order increases

**Table 6:** MSE at different scales vs. age (expressed in days) Spearman correlations (Rho) for control and ADHD group in closed eyes condition (CE)

Scale	Control		ADHD	
	Rho	*p*	Rho	*p*
1	.588	.005	.401	.145
2	.636	.003	.379	.161
3	.608	.003	.374	.159
4	.499	.016	.340	.182
5	.477	.022	.318	.205
6	.473	.022	.315	.194
7	.579	.005	.315	.203
8	.643	.002	.327	.198
9	.719	< .001	.293	.211
10	.721	< .001	.343	.186
11	.732	.001	.348	.188
12	.707	< .001	.299	.207
**13**	**.669**	**.001**	**.303**	**.208**
14	.621	.003	.274	.242
15	.538	.009	.247	.284
16	.446	.031	.211	.365
17	.358	.093	.159	.506
18	.252	.245	.110	.638
19	.081	.722	.030	.886
20	− .061	.773	− .045	.854
21	− .188	.389	− .154	.507
22	− .322	.133	− .247	.295
**23**	**− .462**	**.025**	**− .353**	**.189**
24	− .474	.023	− .401	.133
25	− .536	.009	− .501	.036
26	− .525	.011	− .525	.027
27	− .543	.009	− .569	.034
28	− .558	.007	− .574	.092
29	− .560	.007	− .574	.046
30	− .588	.004	− .557	.022
31	− .616	.003	− .567	.026
32	− .621	.003	− .532	.027
33	− .632	.003	− .549	.022
34	− .684	.001	− .562	.024

P*-values* with FDR correction for multiple comparisons. The MSE values for all the electrodes were collapsed. The limit of the scales is indicated by bold. Notice the transition from positive to negative correlations as scale order increases

**Fig. 4:**
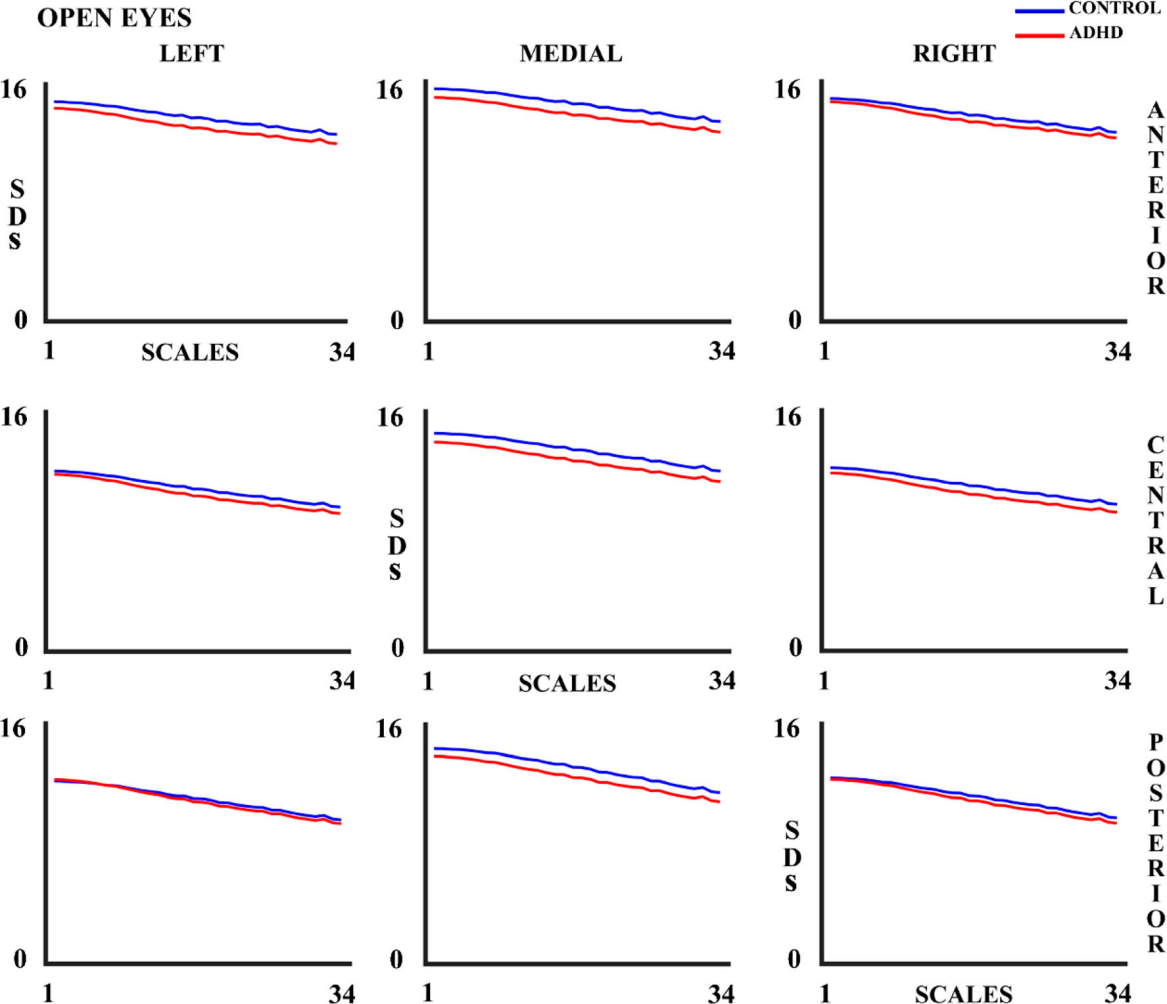
Standard Deviation (SDs) for 34 scales in control and ADHD subjects in the open eyes (OE) experimental condition in all the 9 considered areas. The blue line represents the control group and the red line the ADHD group

**Fig. 5:**
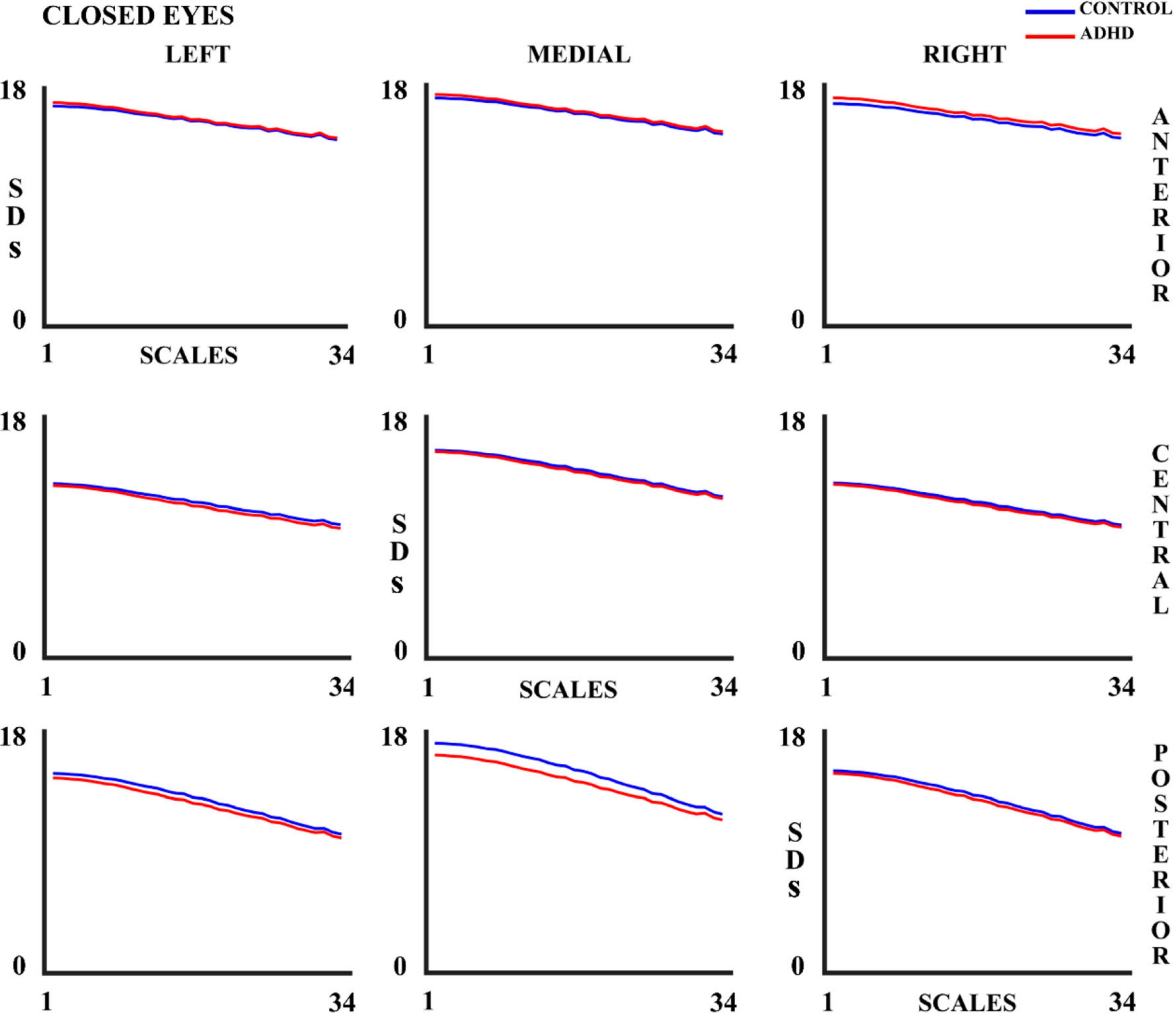
Standard Deviation (SDs) for 34 scales in control and ADHD subjects in the closed eyes (CE) experimental condition in all the 9 considered areas. The blue line represents the control group and the red line the ADHD group

EEG variability (measured as SDs) shows a decrease as the number of scales increases (Figs. 4 and 5), and a decrease with age for both controls and ADHD children (Supplementary tables 1 & 2) in both experimental conditions.

No main or interaction effect was observed among the within-subject factors. All the within-subject significant results for the ANOVAs of OE, CE, and CE&OE are displayed in Supplementary Tables 3 & 4, but they were not further analyzed.

**Fig. 6:**
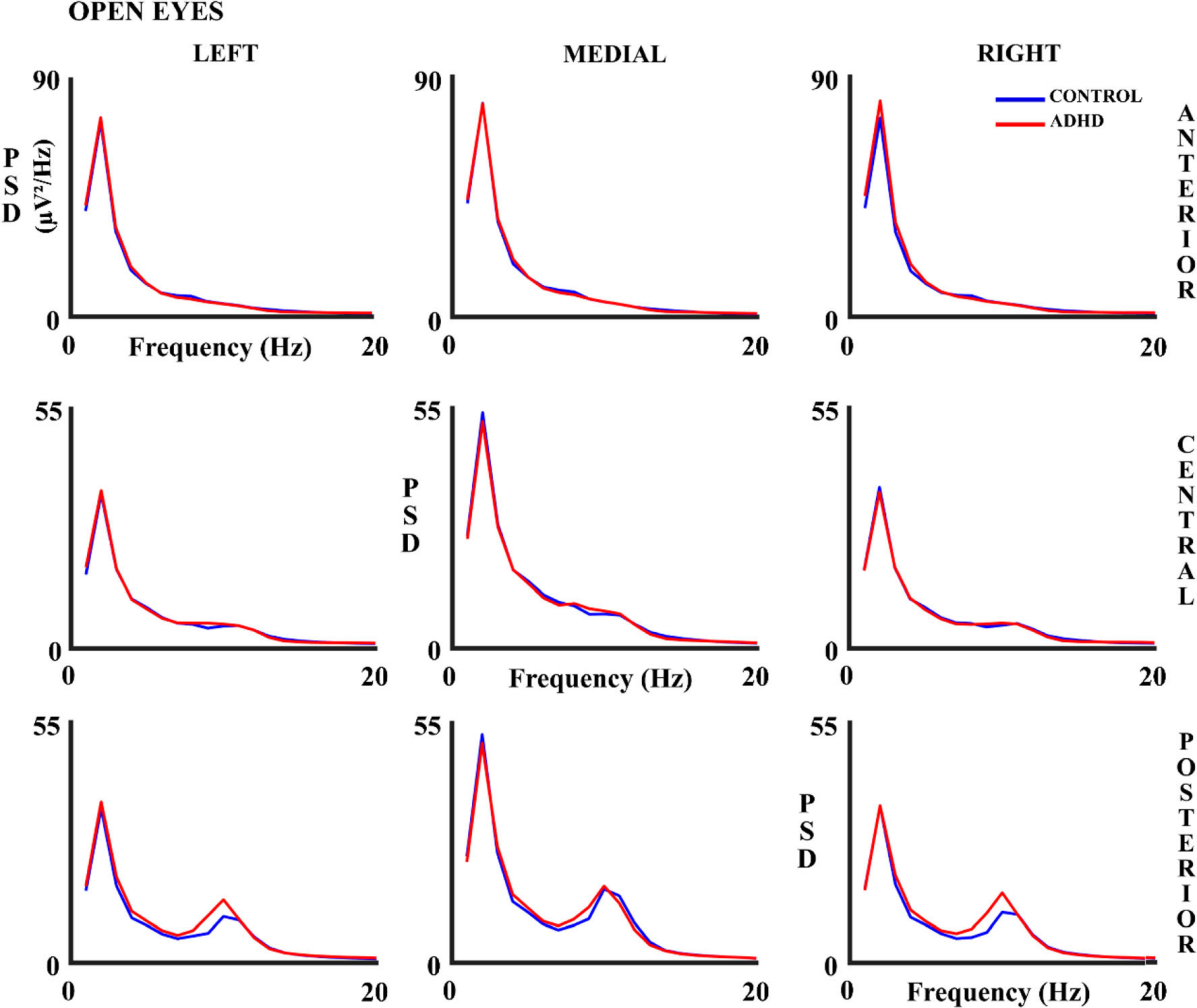
Absolute Power Spectral Density (PSD) in Hz in control and ADHD subjects for the open eyes (OE) experimental condition in all 9 considered areas. The blue line represents the control group and the red line the ADHD group

**Fig. 7:**
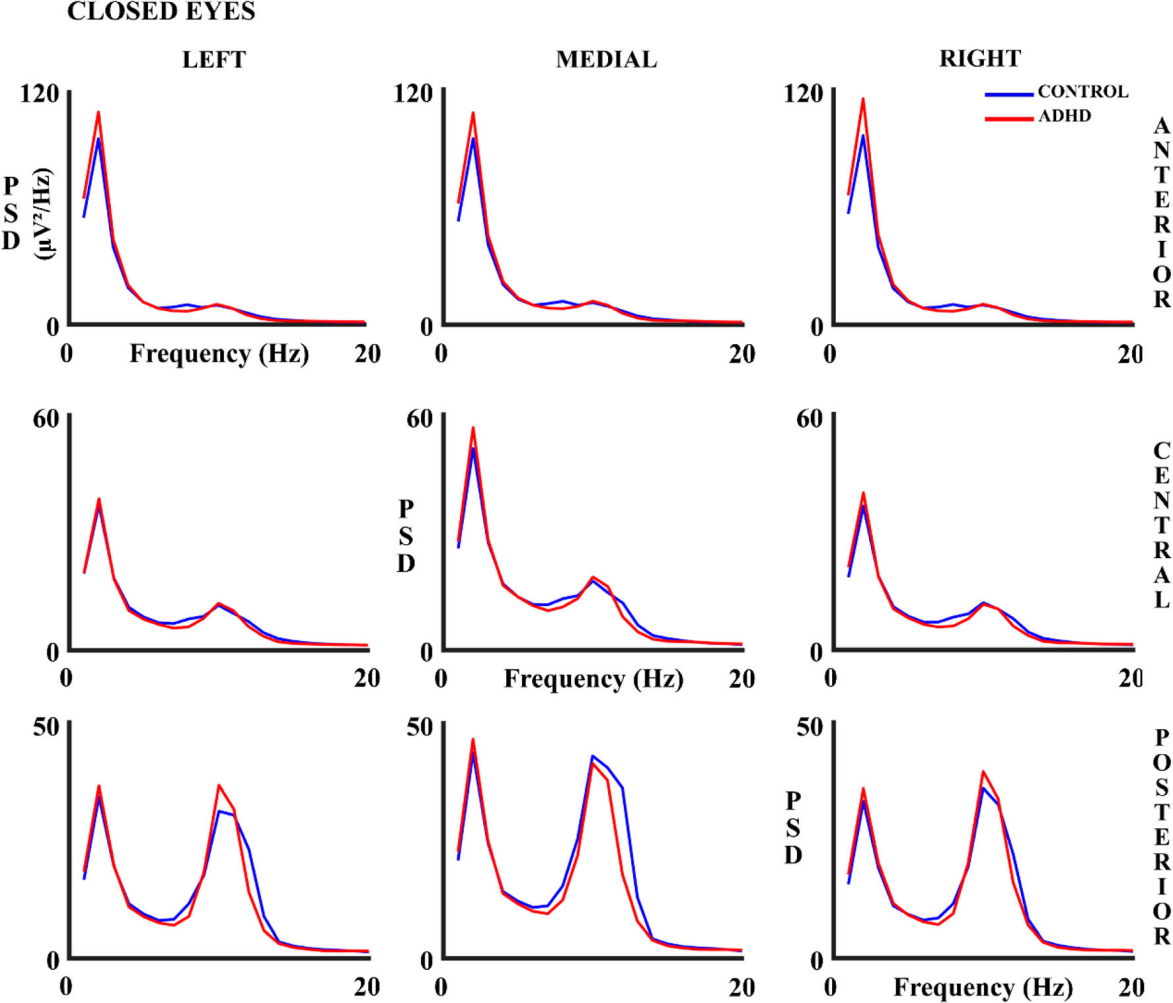
Absolute Power Spectral Density (PSD) in control and ADHD subjects for the closed eyes (CE) experimental condition in all 9 considered areas. The blue line represents the control group and the red line the ADHD group

Figures 6 and 7 show the absolute PSD results in all the analyzed scalp areas for both experimental conditions. The figures suggest that PSD is higher in ADHD children in the delta (CE) and alpha frequency band (OE), mainly in anterior and posterior areas. However, ANOVA results (Supplementary Table 5) show a significant effect solely for the alpha frequency band in the interaction group x lateral x anterior–posterior, both for OE and CE independently. The ANOVA post-hoc analysis showed the interaction of factor group x lateral posterior areas for the OE (F (1.83, 113.7) = 3.41, *p* = 0.040; eta partial squared = 0.052). This interaction was due to a higher difference in alpha PSD between left-posterior areas and medial-posterior areas in controls compared to ADHD (*p* = 0.023). However, in ANOVA post-hoc for CE, there was no interaction with the group in any selected area.

The results of the absolute PSD analysis including experimental conditions (OE & CE) as a within-subjects factor (Supplementary Table 6) show an interaction between groups and experimental conditions in the delta band. The post-hoc analysis (F (1, 46) = 5.39, *p* = 0.025; eta partial squared = 0.105) showed a higher difference of absolute PSD values between CE and OE of ADHD (M = 1.55; SD = 1.17) compared to controls (M = 0.834; SD = 0.936).

The results of Spearman's correlation in both experimental conditions showed a negative correlation of the mean (Supplementary Tables 7 & 8) and standard deviation of PSD with age (Supplementary Tables 9 & 10) in all frequency bands.

Supplementary Table 11 shows the ANOVA results for the CV in OE and CE conditions. Significant differences between the control and ADHD groups were found in the CV for the delta bands in both experimental conditions. In OE CV values are higher in the ADHD group (M = 1.8, SD = 0.069) than in the control group (M = 1.77, SD = 0.067), as well as in CE condition (ADHD (M = 1.83, SD = 0.083), control (M = 1.77, SD = 0.065)). The effect of interactions including the group factor showed anteroposterior differences in the theta and beta bands for OE. However, only the anterior areas of the beta band showed differences between groups when multiple comparisons analysis was performed (*p* = 0.01): higher CV value in the ADHD group (M = 1.69; SD = 0.083) compared to the control group (M = 1.64; SD = 0.043).

The results of the CV analysis including experimental conditions (OE&CE) as a within-subject factor (Supplementary Table 12) show differences between groups in the delta band due to a high amplitude of the CV in ADHD (M = 1.82, SD = 0.055) compared to controls (M = 1.77, SD = 0.054). The theta band shows an interaction between the group and experimental conditions. Namely, the ADHD group (*p* = 0.012) shows a higher CV values in OE (M = 1.67; SD = 0.050) compared to CE (M = 1.64; SD = 0.028), while the control group showed no differences between conditions. The interaction between the group and anteroposterior areas of the theta band shows that in controls the comparisons between the areas are significant: anterior-central (*p* < 0.001), anterior–posterior (*p* = 0.008), and central-posterior (*p* = 0.018) being the CV values of anterior (M = 1.69; SD = 0.052) and posterior areas (M = 1.65; SD = 0.055) higher than central (M = 1.63; SD = 0.039). In the ADHD group, the comparisons between areas were only significant between anterior-central (*p* < 0.001) and anterior–posterior (*p* < 0.001), with the anterior area (M = 1.71; SD = 0.079) presenting a higher CV value than the posterior area (M = 1.63; SD = 0.021), and central area (M = 1.62, SD = 0.023). The OE&CE x anterior–posterior in the beta band group interaction shows differences between controls (M = 1.64; SD = 0.036) and ADHD (M = 1.7; SD = 0.088) in the anterior areas for the OE condition (*p* = 0.005). The other significant interactions in the ANOVA lost their significance when the multiple comparisons corrections (FDR) were performed. The correlation between CV with age was not significant in any group or condition.

The results of the relative PSD analysis (Table 7) showed an interaction between lateral x anterior–posterior x group areas in the alpha band for the OE condition. Post-hoc analysis showed no differences between areas or groups. Analysis including condition, OE & CE (Table 8), as a within-subject factor, showed differences between groups in the delta band. This difference was due to a higher relative PSD value between OE and CE in the control group (M = 2.57, SD = 2.88) compared to the ADHD group (M = 0.53, SD = 3.01).

**Table 7:** Significant results obtained in the ANOVA of the relative PSD with factors: group of subjects, anterior–posterior, and laterality

Bands	OA	OC
Delta (1–2 Hz)	Within-subjects: Anterior–posterior *p* < .001 F = 170.94, gl = [1.33, 82.65], eta partial squared = .734 Laterality x Anterior–posterior *p* < .001 F = 6.04, gl = [3.35, 207.9], eta partial squared = .089	Within-subjects: Laterality *p* < .001 F = 11.96, gl = [1.87, 89.73], eta partial squared = .199 Anterior–posterior *p* < .001 F = 482.79, gl = [1.31, 62.73], eta partial squared = .910 Laterality x Anterior–posterior p < .001 F = 11.54, gl = [3.17, 152.02], eta partial squared = .194
Theta (4–7 Hz)	Within-subjects: Laterality *p* < .001 F = 37.15, gl = [1.96, 121.74], eta partial squared = .375 Anterior–posterior p < .001 F = 45.31, gl = [1.45, 89.7], eta partial squared = .422 Laterality x Anterior–posterior *p* < .001 F = 40.78, gl = [3.68, 228.11], eta partial squared = .397	Within-subjects: Laterality *p* < .001 F = 32.75, gl = [1.91, 91.93], eta partial squared = .406 Anterior–posterior *p* < .001 F = 43.14, gl = [1.39, 66.81], eta partial squared = .473 Laterality x Anterior–posterior *p* < .001 F = 16.35, gl = [3.14, 150.41], eta partial squared = .254
Alpha (8–11 Hz)	Within-subjects: Laterality *p* = .001 F = 7.64, gl = [1.95, 121.09], eta partial squared = .110 Anterior–posterior *p* < .001 F = 101.71, gl = [1.15, 71.62], eta partial squared = .621 Laterality x Anterior–posterior *p* = .043 F = 2.76, gl = [3.01, 186.6], eta partial squared = .043 Laterality x Anterior–posterior x group p = .049* F = 2.67 gl = [3.01, 186.6], eta partial squared = .041	Within-subjects: Laterality *p* < .001 F = 8.96, gl = [1.96, 94.01], eta partial squared = .157 Anterior–posterior *p* < .001 F = 178.6, gl = [1.16, 55.76], eta partial squared = .788 Laterality x Anterior–posterior *p* < .001 F = 6.94, gl = [2.76, 132.29], eta partial squared = .126
Beta (13–20 Hz)	Within-subjects: Laterality *p* < .001 F = 61.12, gl = [1.45, 89.73], eta partial squared = .496 Anterior–posterior *p* < .001 F = 50.39, gl = [1.34, 82.90], eta partial squared = .448 Laterality x Anterior–posterior *p* < .001 F = 15.99, gl = [3.54, 219.48], eta partial squared = .205	Within-subjects: Laterality *p* < .001 F = 23.3, gl = [1.99, 95.63], eta partial squared = .327 Anterior–posterior *p* < .001 F = 39.11, gl = [1.21, 58.2], eta partial squared = .449 Laterality x Anterior–posterior *p* < .001 F = 21.31, gl = [3.59, 172.62], eta partial squared = .307

The ANOVA was computed independently for the open (OE) and closed eyes (CE) conditions. The results in which the factor group was significant as a main or interactive effect are indicated with an asterisk

**Table 8:** Significant results of the ANOVA analysis of the relative PSD values, with factors group of subjects (control and ADHD), laterality, anterior–posterior and open eyes and closed eyes conditions (OE & CE), for each band independently

Delta (1–2 Hz)	Within-subjects: OE-CE p = .001 F = 13.19, gl = [1, 46], eta partial squared = .223 OE-CE x group p = .021* F = 5.75, gl = [1, 46], eta partial squared = .111 Laterality *p* = .005 F = 5.64, gl = [1.95, 89.53], eta partial squared = .109 Anterior–posterior p < .001 F = 315.25, gl = [1.3, 59.69], eta partial squared = .873 OE-CE x Laterality *p* < .001 F = 8.49, gl = [1.97, 90.55], eta partial squared = .156 OE-CE x Anterior–posterior *p* < .001 F = 164.15, gl = [1.47, 67.77], eta partial squared = .781 Laterality x Anterior–posterior *p* < .001 F = 8.34, gl = [3.39, 155.94], eta partial squared = .154
Theta (4–7 Hz)	Within-subjects: OE-CE *p* < .001 F = 78.75, gl = [1, 46], eta partial squared = .631 Laterality *p* < .001 F = 46.79, gl = [1.84, 84.48], eta partial squared = .504 Anterior–posterior *p* < .001 F = 51.97, gl = [1.36, 62.43], eta partial squared = .530 OE-CE x Anterior–posterior *p* = .016 F = 4.96, gl = [1.55, 71.51], eta partial squared = .097 Laterality x Anterior–posterior *p* < .001 F = 27.5, gl = [3.46, 159.14], eta partial squared = .374 OE-CE x laterality x anterior–posterior *p* = .009 F = 3.7, gl = [3.59, 165.49], eta partial squared = .074
Alpha (8–11 HZ)	Within-subjects: OE-CE p < .001 F = 71.6, gl = [1, 46], eta partial squared = .609 Laterality *p* < .001 F = 10.81, gl = [1.98, 91.19], eta partial squared = .190 Anterior–posterior *p* < .001 F = 144.49, gl = [1.15, 52.68], eta partial squared = .759 OE-CE x Anterior–posterior *p* < .001 F = 98.35, gl = [1.27, 58.25], eta partial squared = .681 Laterality x Anterior–posterior *p* < .001 F = 6.61, gl = [2.99, 137.78], eta partial squared = .126 OE-CE x Laterality x Anterior–posterior *p* = .005 F = 4.93, gl = [2.59, 119.24], eta partial squared = .097
Beta (13–20 Hz)	Within-subjects: OE-CE *p* = .007 F = 7.93, gl = [1, 46], eta partial squared = .147 Laterality p < .001 F = 41.75, gl = [1.66, 76.29], eta partial squared = .476 Anterior–posterior *p* < .001 F = 46.99, gl = [1.25, 57.58], eta partial squared = .505 OE-CE x Laterality *p* < .001 F = 18.97, gl = [1.58, 72.74], eta partial squared = .292 Laterality x Anterior–posterior *p* < .001 F = 22.89, gl = [3.36, 154.52], eta partial squared = .332

All significant results are displayed. The results in which the factor group was significant as a main or interactive effect are indicated with an asterisk

Results of Spearman Correlations for both experimental conditions OE (Table 9) and CE (Table 10) show that relative PSD decreases in the slow frequency bands and increases in the fast frequency bands with age. A greater correlation (in absolute values) was observed for controls (M = 0.435, SD = 0.181) when compared to ADHD subjects (M = 0.325, SD = 0.118) in the CE condition (*p* = 0.029) (based on a t-test between the absolute correlation values).

**Table 9:** Relative PSD vs. age (in days) Spearman correlations for control and ADHD group in open eyes condition (OE)

Frequencies (Hz)	Control		ADHD	
	R	*p*	R	*p*
1	− .409	.059	− .204	.325
2	− .547	.005	− .032	.861
3	− .282	.157	− .101	.608
4	− .079	.667	− .176	.391
5	− .207	.282	− .278	.176
6	− .319	.117	− .382	.091
7	− .243	.212	− .317	.141
8	− .289	.156	− .291	.178
9	− .249	.210	− .319	.151
10	− .102	.609	− .125	.550
11	.358	.082	.266	.188
12	.737	< .001	.377	.086
13	.814	< .001	.529	.021
14	.789	< .001	.546	.028
15	.644	< .001	.502	.025
16	.477	.021	.447	.054
17	.362	.085	.429	.059
18	.327	.114	.386	.100
19	.369	.086	.324	.158
20	.405	.056	.281	.183

*P-values* with FDR correction for multiple comparisons. Notice the transition from positive to negative correlations as frequency increases

**Table 10:** Relative PSD vs. age (in days) Spearman correlations for control and ADHD group in closed eyes condition (CE)

Frequencies (Hz)	Control		ADHD	
	R	*p*	R	*p*
1	− .375	.101	− .135	.544
2	− .409	.087	− .259	.279
3	− .476	.043	− .341	.239
4	− .398	.089	- .323	.231
5	− .478	.048	− .587	.049
6	− .547	.026	− .533	.068
7	− .345	.122	− .519	.057
8	− .299	.172	− .357	.268
9	− .381	.102	− .130	.534
10	− .042	.841	.216	.331
11	.480	.054	.382	.304
12	.765	< .001	.333	.231
13	.805	< .001	.351	.246
14	.709	< .001	.372	.270
15	.533	.027	.322	.213
16	.289	.178	.292	.259
17	.221	.302	.248	.272
18	.336	.126	.265	.286
19	.370	.099	.256	.269
20	.445	.059	.289	.247

*P-values* with FDR corrections for multiple comparison. Notice the transition from positive to negative correlations as frequency increases

**Fig. 8:**
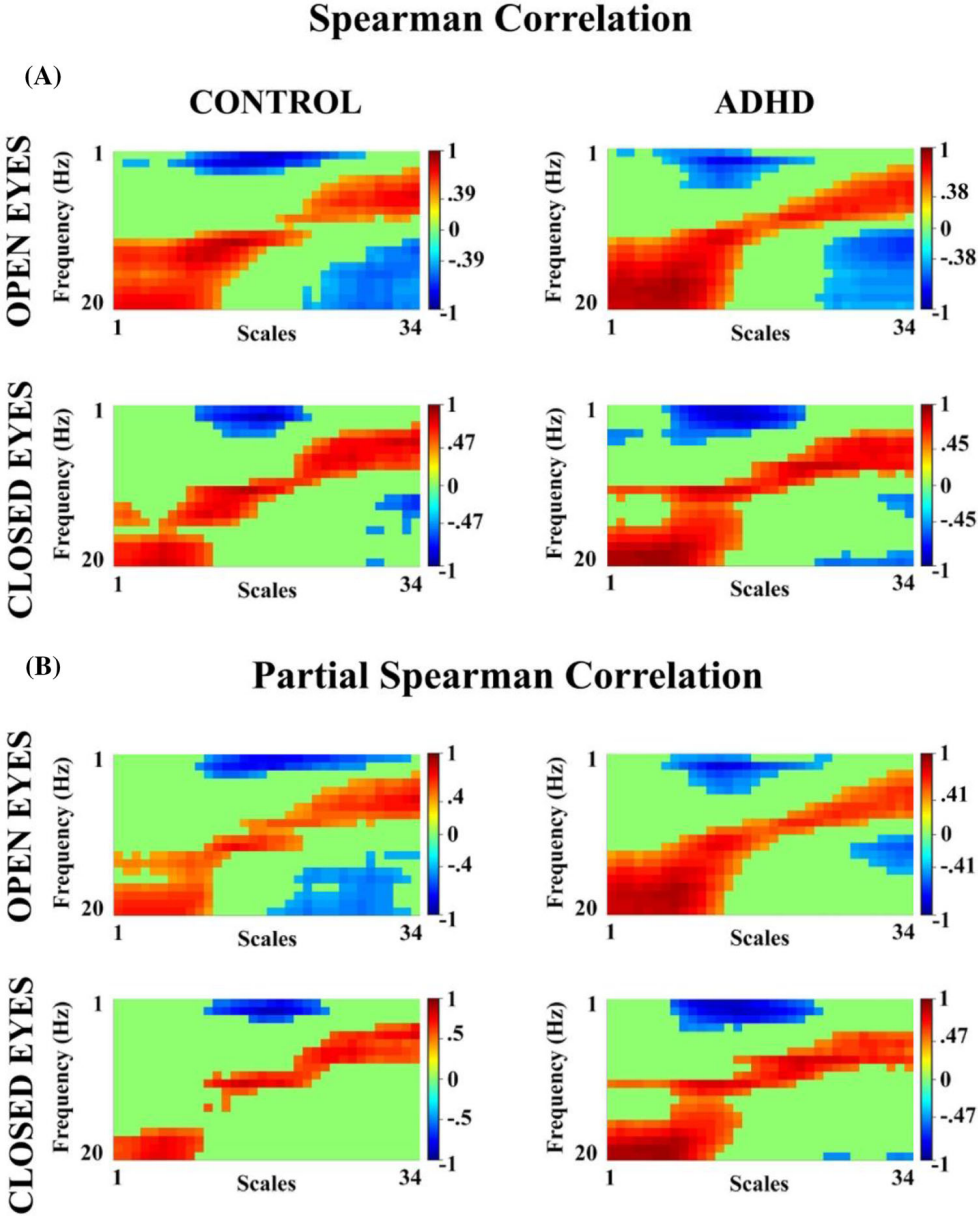
**A** Spearman Correlation between MSE (34 scales) and relative PSD (1–20 Hz) in control and ADHD subjects for both experimental conditions** B** Partial Spearman correlation, controlling for age (in days) in open and closed eye conditions for both groups of subjects. The cutoff value for each correlation, being significant when corrected by FDR, is indicated in the graphs. The red color represents positive correlations and the blue one represents negative correlations

The results of the correlations between the relative PSD (1–20 Hz) and the scales (34 scales) (Fig. 8A) show significant positive correlations (corrected by FDR) in OE (controls (cutoff for Rho > 0.39, *p* < 0.025), ADHD (cutoff for Rho > 0.39, *p* < 0.027)), and CE (controls (cutoff for Rho > 0.47, *p* < 0.017), ADHD (cutoff for Rho > 0.45, *p* < 0.022)), as well as significant negative correlations in both OE and CE conditions. The significant positive correlations were between the high frequencies (beta) and fine scales (1–13 scales) and low frequencies (delta, theta) with coarse scales (24–34 scales), whilst for negative correlations high frequencies vs coarse scales and low frequencies vs medium scales (14–23 scales) were significant. These results support the relationship between both metrics. Partial correlations were calculated to control for the effect of the age of the subjects, and significant correlations between MSE and relative PSD were found. A similar pattern was observed in correlations when age was controlled (Fig. 8B), when compared to non age-controlled correlation (Fig. 8A), suggesting a low effect of maturation on these metrics in both groups and conditions.

Finally, in the ADHD (OE and CE) group, Spearman correlations were computed between the different measures analyzed: MSE, SDs, absolute PSD (mean and CV), and relative PSD with the behavioral measures of impulsivity and inattention of the DuPaul questionnaire (DuPaul et al. 1998). No significant results were found.

## Discussion

We analyzed a group of children with ADHD compared to an age and gender-matched normative group under experimental conditions of OE and CE using MSE, SDs, and PSD (absolute (mean, SDp and CV) and relative (mean)) methods to observe possible alterations of brain rhythms and evaluate the complexity and variability of the neural EEG. We hypothesized that control children would present differences in EEG MSE and variability compared to ADHD children, and would show a strong correlations between all EEG metrics and age.

For the correlational analysis of the different EEG metrics with age, many studies support the increase of endogenous cortical dynamic complexity with age across lower frequency bands (McIntosh et al. 2008, 2010; Garrett et al. 2013; Miskovic et al. 2016; Van Noordt & Willoughby 2021). Such increase would be a consequence of network functional reconfigurations that shift from more stable to more variable states with age. Brain network functional organization across brain maturation would be related to MSE (McIntosh et al. 2008, 2010). However, our study indicates a limitation of the positive relationship of age across scales in both OE and CE conditions. The results show that in the 34 temporal scales evaluated in resting state there is an inversion (positive to negative) of the relationship between MSE with age, as a function of the scales analyzed. The inversion from the positive to a negative relationship between MSE and age occurs in the coarser scales for both groups: controls and ADHD. This is in agreement with results found by Szostakiwskyj et al. (2017) who suggest that the increase of the MSE with age becomes gradually weaker as the number of scales increases reaching a reversal of the relationship at coarse time scales. As such, both Szostakiwskyj et al. (2017) study and our study support the idea that, in both groups (controls and ADHD), local information processing increases throughout the brain with age (positive MSE-age relationship on fine scales), and that, at later ages in development, interactions between long-range neural populations would be reduced (negative MSE-age relationship on medium and coarse scales) given the low-frequency dominance in coarser scales (Szostakiwskyj et al. 2017).

Similarly, previous studies with PSD suggest that the increase in the high-frequency bands with age (Segalowitz et al. 2010) is related to greater processing of local information; while the decrease in low frequencies corresponds to a decrease in long-range communication with other neuronal populations (Gasser et al. 1988; Von Stein et al. 2000; McIntosh et al. 2008; Cragg et al. 2011). MSE studies have shown that the scales contain information regarding the frequencies, with fine scales having information of all frequencies, and the coarse scales with the low frequencies (McIntosh et al. 2008, 2010; Szostakiwskyj et al. 2017; Bosl et al. 2022). Our results support these statements showing a strong relationship between the relative PSD and the MSE with age, given by an increase in the high-frequency bands and fine scales, and a decrease in the low frequency bands and coarse scales with age, indirectly involving a connectivity refinement pattern. Likewise, an indirect relationship between the establishment of a small-world topology during development, characterized by an increase in clustering coefficients and path lengths with age (Boersma et al. 2011), and the increase and decrease of the MSE and relative PSD could occur. EEG studies with the normodevelopment population suggest that there is an increase in small-world topology during development as suggested by increased clustering and path length coefficients with age (Boersma et al. 2011; Smit et al. 2012; Vértes & Bullmore 2015). The oscillatory frequencies have also been related to connectivity in the brain; thus, low frequency bands reflecting global activity of brain, and high frequency bands reflecting its local connectivity (Li et al. 2016). Present results showed a more consistent pattern of inversion of the MSE (from fine to coarse scales: higher frequencies to lower frequencies) and relative PSD (from low to high frequencies) with age in controls than in ADHD, suggesting that the establishment of focally segregated and long-range refined integrated topology in neural networks with maturation occurs more clearly in controls than in ADHD.

Studies investigating mean PSD throughout development have shown generalized decreases in absolute power in all brain rhythms, and decreases in low-frequency bands and increases in high rhythms in relative power (Dustman et al. 1999; McIntosh et al. 2008; Barry et al. 2009; Segalowitz et al. 2010; Rodríguez-Martínez et al. 2012, 2017, 2020). The results of the present study confirm the negative relationship of absolute PSD with age in both groups analyzed and for OE and CE, validating this EEG parameter as a robust biomarker of EEG maturation (Gasser et al. 1988; Segalowitz et al. 2010; Rodríguez-Martínez et al. 2012; Miskovic et al. 2015). In this sense, the decrease in PSD with age could be explained by the process of synaptic pruning in which the most stable neural connections are maintained, and the rest are pruned, increasing the efficiency of neural transmission (Whitford et al. 2007). The inversion of the correlation of the age with the relative PSD, from negative correlations in low frequencies to positive correlations, determines a higher contribution of high frequencies to the EEG power as maturation progresses (Segalowitz et al. 2010). On the other hand, the main differences in PSD (absolute and relative) between the groups were observed in the delta and alpha frequency bands. For the delta band, the values of the difference between the conditions (CE-OE) of the absolute PSD were higher for the ADHD group, in contrast to the relative PSD, where these values were higher for the control group. Regarding the alpha band, the differences between groups in absolute and relative PSD were found in the OE condition. However, only the absolute PSD of the control group shows higher difference values, between the posterior-left and posterior-medial areas compared to ADHD. Although the present report did not find increased mean PSD of ADHD with respect to controls in low-frequency bands, differences were obtained as effects of interactions of the group factor with the experimental condition. The latter could be explained by the relatively low subjects’ sample and the variability of ADHD children’s PSD. Similarly, it has been reported that theta was increased in 60% of subjects and decreased in 40% of them (Clarke et al. 2011).

The aforementioned high correlation between relative PSD and MSE, even when the partial correlation is controlled by age, suggests a functional dependency between these two metrics, as was suggested by Bosl et al. (2022). Similarly, previous studies which have analyzed this relationship have found a similar pattern in the relationship between MSE and relative PSD for low and medium scales (high frequencies) (McIntosh et al. 2008; Kosciessa et al. 2020; Van Noord & Willoughby, 2021). Considering such relationship between scales and frequency bands (Bosl et al. 2022) and the use of a sampling rate of 512 Hz and 34 scales (the higher scale related to frequencies ≤ 7.73 Hz) in the present study, the coarsest scales would be equivalent to delta and theta bands, the medium scales to alpha and low beta, and fine scales to beta. It is important to note that due to the high pass filter the gamma band or above it is not considered. Nevertheless, MSE is sensitive to linear and non-linear temporal dependencies in EEG signal irregularities, while PSD is only to linear changes (Van Noordt & Willoughby 2021). The fact that in the present report no clear direct differences between relative PSD of controls and ADHD were found (significant differences appeared as an interaction of factor effects), while these differences were obtained in MSE as a main group factor effect, suggests that maturational differential effects between control and ADHD occur in non-linear temporal dependencies in the analyzed samples of ADHD and controls.

Our results of brain signal variability across scales (SDs) and spectral power (SDp) are complementary to the MSE. While MSE in the whole brain increases and decreases with increasing number of scales (from higher to lower frequencies bands) and age, SDs and SDp decrease in both scales and frequencies (1–20 Hz) with age, and in both experimental conditions for both groups (controls-ADHD). These results show a decreased variability of EEG related to the reduction of EEG absolute PSD with age, also observed in the present results. The decrease in absolute spectral power with age accompanied by EEG reduced variability with age (SDs and SDp), would be a consequence of neural pruning processes that would reduce EEG amplitude (Whitford et al. 2007). However, calculations of SDs and SDp differ from MSE in the fundamental aspect that MSE is computed considering a normalization of the similarity limit (r) parameter by the EEG standard deviation of the trial, and in that regard, MSE would be more related computationally to the variability of the CV across trials of the different frequency bands, given that in the CV the standard deviation of the EEG is normalized by the mean. The CV did not show modulation by age neither in controls nor in ADHD, but the mean comparisons of the CV across groups showed an increased CV in delta and beta rhythms for ADHD with respect to controls, particularly clear in anterior areas. The latter results suggest that spontaneous EEG is more variable in ADHD than in controls. The higher variability in behavior and neural responses of ADHD subjects with respect to controls has been previously observed (Castellanos et al. 2009). Therefore, the present results add evidence to more variable activation levels in ADHD subjects compared to controls while a higher complexity is obtained in controls with respect to ADHD.

EEG variability as measured by SDs, SDp, and CV could be considered as a first-order measurement of variability, which does not fully incorporate the presence of organized patterns of different temporal scales. MSE incorporates variability and organization of time series and is therefore well suited to analyze physiological signals (Garrett et al. 2013; McIntosh et al. 2010). As such, complexity analysis of several psychiatric and psychological impairments has shown EEG abnormalities (Catarino et al. 2011; Bosl et al. 2011, 2017; Chu et al. 2017; Takahashi et al. 2013; Papaioannou et al. 2021). In developmental disorders such as ADHD, particularly, not many studies have been conducted, leading to inconsistencies in the results. As an example, Fernández et al. (2009) showed a decrease in complexity (Lempel–Ziv complexity (LZC)) with age in the ADHD group compared to an increase in the control group; Rezaeezadeh et al. (2020), using MSE, suggests that children with ADHD present lower values of complexity than a normative group; and Li et al. (2016) indicates higher values of complexity for ADHD subjects compared to controls in delta and theta rhythms and lower complexity in alpha rhythms. Our findings show an increase in complexity across increasingly coarser time scales, in both groups analyzed, for both conditions OE and CE. The group comparison showed higher MSE values for controls compared to ADHD, suggesting an increasingly variable and less predictable neural signal (Garrett et al. 2013) across different temporal scales (Szostakiwskyj et al. 2017) in the resting state. Recently, functional near-infrared spectroscopy (fNIRS) study shows that children with ADHD have reduced complexity in primary and higher-order functional brain networks, such as the default mode network, fronto-parietal, attentional, and visual networks (Hu et al. 2021). Therefore, the higher complexity observed in the control group could reflect increased information processing capacity (Szostakiwskyj et al. 2017), greater maturation of stable behavioral responses (McIntosh et al. 2008; Misic et al. 2010), and effective adaptation to environmental uncertainty (Grady & Garrett 2018), highlighting a difference in these processes with children with ADHD. Therefore, the present results indicate that ADHD children present more brain rhythms variability (as indicated by CV), but a lower EEG complexity (as indicated by MSE) than controls.

The present results support the maturational delay model due to the reduced complexity in ADHD subjects compared to controls, given that these children present MSE values below biological age (Szostakiwskyj et al. 2017). Interestingly, and as indicated before, ADHD showed a less clear pattern (lower correlation MSE vs. age) of the increase for the fine scales and the decrease in the coarse scales with age than controls. A similar developmental pattern of ADHD (lower correlation relative PSD vs age) was also shown by relative PSD of decreasing relative power in low frequencies and increasing in higher frequencies with age. The latter results suggest not only a maturational delay but also a differential developmental trajectory to reach maturation as indicated by inter-group significant differences of MSE and relative PSD correlations with age.

The decreased complexity and higher variability of EEG in ADHD compared to control children as well as the interactive effects in PSD low-frequency bands could suggest that children with ADHD display abnormal functioning at the neural level for their biological age compared to normal children (Shaw et al. 2007; Narr et al. 2009; Giertuga et al. 2017; Saad et al. 2018; Rodríguez-Martínez et al. 2020). Nevertheless, other studies have reported increases in complexity and/or variability (Li et al. 2016) as well as increases in PSD in low frequencies in OE in the ADHD group (Sohn et al. 2010; Nazari et al. 2011), so further research is still needed to achieve more consistent agreements. In general, these differences could be affected by variability in ADHD typology, medication administration, and/or comorbidities with the disorder.

Consequently, it is pertinent to mention the limitations of this study, such as the number of subjects analyzed, and the small number of women included in the study. This could be explained by the higher prevalence of the disorder in the male population, in this matter some studies have demonstrated the existence of gender differences in the EEG in people with ADHD. With the EEG of women being more homogeneous and less aberrant than men with the disorder (Clarke et al. 2020), which could be affecting the symptomatology, and in turn, leading the low levels of diagnosis. Therefore, in future research, it would be recommendable to include a greater number of women in the studies. Although a minimal number of 50 samples was suggested for a reliable estimation of MSE, and the present report uses only 31 samples for the coarsest scale, MSE computation is considered accurate based on the obtained results of pattern inversion in the correlation of MSE with age. Nevertheless, future studies would include a longer period of resting-state recording which would permit to segment EEG data in longer epochs and to have enough number of trials to compute more reliable MSE estimations.

## Conclusions

The present study showed a decrease of MSE as well as an increase in EEG variability (CV) in ADHD compared to controls. These results suggest a less stable neural environment and a lower capacity to adapt to new situations in ADHD with respect to controls. The results also indicate, that in both groups an increase in MSE with age occurs for fine scales while a decrease occurs for coarse scales. The latter results suggest that during development strengthening of local connections, while refinement of long-range connections occurs, which is compatible with the establishment of a small-world network topology across development, more homogenously established in controls than in ADHD.

## Supplementary Information

Below is the link to the electronic supplementary material.

Supplementary file1 (DOCX 34 KB)
