# Supplementary material for: Multiscale entropy of ADHD children during resting state condition
Source: Cogn Neurodyn. 2022 Aug 30;17(4):869–91. doi: 10.1007/s11571-022-09869-0 (PMC10374506; doi:10.1007/s11571-022-09869-0)
Supplement: Supplementary file 1 — Supplementary file1 (DOCX 34 KB) [file 11571_2022_9869_MOESM1_ESM.docx]

**Supplementary Tables**

| **Supplementary Table 1.** | | | | |
| --- | --- | --- | --- | --- |
| Standard Deviation (SDs) vs. age (expressed in days) Spearman correlations (Rho) for control and ADHD group in open eyes condition (OE) in all the different scales. The standard deviation of all electrodes are collapsed. *P-values* with FDR correction for multiple comparisons. The thick lines indicate the limit of the different scales (fine, medium and coarse scales). Notice the negative correlation for all the scales. | | | | |
| **Scales** | **CONTROL** | | **ADHD** | |
|  | **Rho** | ***p*** | **Rho** | ***p*** |
| 1 | -.725 | < .001 | -.798 | < .001 |
| 2 | -.725 | < .001 | -.795 | < .001 |
| 3 | -.725 | < .001 | -.801 | <. 001 |
| 4 | -.729 | < .001 | -.797 | < .001 |
| 5 | -.729 | < .001 | -.797 | < .001 |
| 6 | -.731 | < .001 | -.798 | < .001 |
| 7 | -.731 | < .001 | -.797 | < .001 |
| 8 | -.731 | < .001 | -.793 | < .001 |
| 9 | -.728 | < .001 | -.797 | < .001 |
| 10 | -.731 | < .001 | -.793 | < .001 |
| 11 | -.734 | < .001 | -.795 | < .001 |
| 12 | -.747 | < .001 | -.793 | < .001 |
| **13** | **-.747** | **< .001** | **-.793** | **< .001** |
| 14 | -.745 | < .001 | -.791 | < .001 |
| 15 | -.750 | < .001 | -.793 | < .001 |
| 16 | -.757 | < .001 | -.793 | < .001 |
| 17 | -.761 | < .001 | -.793 | < .001 |
| 18 | -.761 | < .001 | -.790 | < .001 |
| 19 | -.766 | < .001 | -.798 | < .001 |
| 20 | -.766 | < .001 | -.800 | <.001 |
| 21 | -.769 | < .001 | -.800 | < .001 |
| 22 | -.777 | < .001 | -.800 | < .001 |
| **23** | **-.777** | **< .001** | **-.800** | **< .001** |
| 24 | -.780 | < .001 | -.800 | < .001 |
| 25 | -.785 | < .001 | -.802 | < .001 |
| 26 | -.782 | < .001 | -.802 | < .001 |
| 27 | -.790 | < .001 | -.808 | < .001 |
| 28 | -.791 | < .001 | -.806 | < .001 |
| 29 | -.797 | < .001 | -.806 | < .001 |
| 30 | -.791 | < .001 | -.808 | < .001 |
| 31 | -.797 | < .001 | -.808 | < .001 |
| 32 | -.799 | < .001 | -.812 | < .001 |
| 33 | -.799 | < .001 | -.812 | < .001 |
| 34 | -.799 | < .001 | -.812 | < .001 |

| **Supplementary Table 2.** | | | | |
| --- | --- | --- | --- | --- |
| Standard Deviation (SDs) vs. age (expressed in days) Spearman correlations (Rho) for control and ADHD group in closed eyes condition (CE) in all the different scales. The standard deviations of all electrodes are collapsed. *P-values* with FDR correction for multiple comparisons. The thick lines indicate the limit of the different scales (fine, medium, and coarse scales). Notice the negative correlation for all the scales. | | | | |
| **Scales** | **CONTROL** | | **ADHD** | |
|  | **Rho** | ***p*** | **Rho** | ***p*** |
| 1 | -.611 | .001 | -.627 | .001 |
| 2 | -.611 | .001 | -.627 | .001 |
| 3 | -.611 | .001 | -.615 | .001 |
| 4 | -.611 | .001 | -.615 | .001 |
| 5 | -.611 | .001 | -.620 | .001 |
| 6 | -.621 | .001 | -.625 | .001 |
| 7 | -.619 | .001 | -.632 | < .001 |
| 8 | -.619 | .001 | -.632 | < .001 |
| 9 | -.643 | < .001 | -.647 | < .001 |
| 10 | -.642 | < .001 | -.648 | < .001 |
| 11 | -.650 | < .001 | -.657 | < .001 |
| 12 | -.650 | < .001 | -.664 | < .001 |
| **13** | **-.664** | **< .001** | **-.677** | **< .001** |
| 14 | -.673 | < .001 | -.679 | < .001 |
| 15 | -.683 | < .001 | -.679 | < .001 |
| 16 | -.687 | < .001 | -.674 | < .001 |
| 17 | -.691 | < .001 | -.679 | < .001 |
| 18 | -.692 | < .001 | -.679 | < .001 |
| 19 | -.695 | < .001 | -.679 | < .001 |
| 20 | -.695 | < .001 | -.673 | < .001 |
| 21 | -.699 | < .001 | -.680 | < .001 |
| 22 | -.702 | < .001 | -.680 | < .001 |
| **23** | **-.701** | **<.001** | **-.683** | **< .001** |
| 24 | -.702 | < .001 | -.694 | < .001 |
| 25 | -.702 | < .001 | -.695 | < .001 |
| 26 | -.702 | < .001 | -.708 | < .001 |
| 27 | -.735 | < .001 | -.715 | < .001 |
| 28 | -.752 | < .001 | -.731 | < .001 |
| 29 | -.768 | < .001 | -.718 | < .001 |
| 30 | -.784 | < .001 | -.718 | < .001 |
| 31 | -.796 | < .001 | -.732 | < .001 |
| 32 | -.804 | < .001 | -.732 | < .001 |
| 33 | -.793 | < .001 | -.730 | < .001 |
| 34 | -.793 | < .001 | -.734 | .001 |

| **Supplementary Table 3.** | |
| --- | --- |
| Significant results of the ANOVA analysis of Standard Deviation (SDs) values, with factors group of subjects (control and ADHD), scales (fine, medium, and coarse), laterality, and anterior-posterior, for open eyes (OE) and closed eyes (CE) conditions, independently. All significant results are displayed. The group factor was not significant. | |
| **OPEN EYES** | **Within-subjects:**  Scales p < .001*****  F =599.24, gl = [1.12, 69.71], eta partial squared = .906  Laterality p < .001*  F = 314.82, gl = [1.76, 108.98], eta partial squared = .835  Anterior-posterior p < .001*****  F = 100.68, gl = [1.42, 88.33], eta partial squared = .619  Scales x Laterality p= .001*****  F = 9.25, gl = [1.48, 91.92], eta partial squared = .130  Scales x Anterior-posterior p < .001*****  F = 46.37, gl = [1.5, 93.15], eta partial squared = .428  Laterality x Anterior-posterior p < .001*****  F = 69.66, gl = [2.37, 147.02], eta partial squared = .529  Scales x Laterality x Anterior-posterior p < .001*  F = 9.38, gl = [3.41, 211.54], eta partial squared = .131 |
| **CLOSED EYES** | **Within-subjects:**  Scales p < .001*****  F =443.25, gl = [1.03, 49.49], eta partial squared = .902  Laterality p < .001*****  F = 192.67, gl = [1.74, 83.7], eta partial squared = .801  Anterior-posterior p < .001*****  F = 77.16, gl = [1.27, 60.97], eta partial squared = .616  Scales x Laterality p < .001*****  F = 23.8, gl = [1.38, 66.36], eta partial squared = .331  Scales x Anterior-posterior p < .001*****  F = 96.7, gl = [1.08, 51.78], eta partial squared = .668  Laterality x Anterior-posterior p < .001*****  F = 49.44, gl = [2.24, 107.31], eta partial squared = .507  Scales x Laterality x Anterior-posterior p = .002  F = 6.79, gl = [1.95, 93.42], eta partial squared = .124 |

| **Supplementary Table 4.** | |
| --- | --- |
| Significant results of the ANOVA analysis of Standard Deviation (SDs) values, with factors group of subjects (control and ADHD), scales (fine, medium, and coarse), laterality, anterior-posterior, and open eyes and closed eyes conditions (OE & CE). All significant results are displayed. The group factor was not significant. | |
| **Within-subjects** | OE-CE p < .001*  F =183.73, gl = [1, 46], eta partial squared = .800  Scales p < .001*****  F =477.05, gl = [1.07, 49.26], eta partial squared = .912  Laterality p < .001*  F = 248.42, gl = [1.73, 79.73], eta partial squared = .844  Anterior-posterior p < .001*****  F = 93.54, gl = [1.34, 61.74], eta partial squared = .670  OE-CE x Scales p< .001*  F = 71.71, gl = [1.10, 50.71], eta partial squared = .609  Scales x Laterality p < .001*****  F = 17.06, gl = [1.52, 70.02], eta partial squared = .271  OE-CE x Scales x Laterality p< .001*  F = 17.27, gl = [1.64, 75.62], eta partial squared = .273  OE-CE x Anterior-posterior p< .001*  F = 19.94, gl = [1.4, 64.06], eta partial squared = .302  Scales x Anterior-posterior p < .001*****  F = 86.06 gl = [1.15, 53.06], eta partial squared = .652  OE-CE x Scales x Anterior-posterior p< .001*  F = 73.95, gl = [1.12, 51.46], eta partial squared = .617  Laterality x Anterior-posterior p < .001*****  F = 53.31, gl = [2.07, 95.29], eta partial squared = .537  OE-CE x Laterality x Anterior-posterior p= .001*  F = 6.72, gl = [2.48, 114.16], eta partial squared = .127  Scales x Laterality x Anterior-posterior p < .001*  F = 7.44, gl = [2.39, 110.02], eta partial squared = .139  OE-CE x Scales x Laterality x Anterior-posterior p= .041  F = 3.17, gl = [2.19, 101.04], eta partial squared = .065 |

| **Supplementary Table 5.** | | |
| --- | --- | --- |
| Significant results obtained in the ANOVA analysis of the mean of absolute PSD values, with factors: group of subjects, anterior-posterior, and laterality. The ANOVA was computed independently for the open (OE) and closed eyes (CE) conditions. The results in which the factor group was significant as a main or interactive effect are indicated in bold. | | |
| **BANDS** | **OA** | **OC** |
| **DELTA**  **(1-2 Hz)** | **Within-subjects:**  Laterality p< .001  F=195.5, gl= [1.98, 122.74], eta partial squared= .759  Anterior-posterior p< .001  F= 235.55, gl= [1.30, 80.59], eta partial squared= .792  Laterality x Anterior-posterior p< .001  F= 53.49, gl= [3.49, 216.36], eta partial squared= .463 | **Within-subjects:**  Laterality p< .001  F=85.84, gl= [1.89, 90.78], eta partial squared= .641  Anterior-posterior p< .001  F= 359.29, gl= [1.25, 60.16], eta partial squared= .882  Laterality x Anterior-posterior p< .001  F= 48.49, gl= [3.2, 153.71], eta partial squared= .503 |
| **THETA**  **(4-7 Hz)** | **Within-subjects:**  Laterality p< .001  F=817.7, gl= [1.93, 119.41], eta partial squared= .930  Anterior-posterior p< .001  F=111.53, gl= [1.45, 89.6], eta partial squared= .643  Laterality x Anterior-posterior p< .001  F= 201.57, gl= [3.51, 217.95], eta partial squared= .765 | **Within-subjects:**  Laterality p< .001  F=539.26, gl= [1.9, 95.59], eta partial squared= .918  Anterior-posterior p< .001  F=44.41, gl= [1.28, 61.3], eta partial squared= .481  Laterality x Anterior-posterior p< .001  F= 105.69, gl= [2.9, 138.82], eta partial squared= .688 |
| **ALPHA**  **(8-11 Hz)** | **Within-subjects:**  Laterality p< .001  F=447.71, gl= [1.96, 121.59], eta partial squared= .878  Anterior-posterior p< .001  F= 25.6, gl= [1.22, 75.4], eta partial squared= .292  Laterality x Anterior-posterior p< .001  F= 88.64, gl= [2.77, 171.79], eta partial squared= .588  **Laterality x Anterior-posterior x group p= .032***  F= 3.09 gl= [2.77, 171.79], eta partial squared= .047 | **Within-subjects:**  Laterality p< .001  F=334.3, gl= [1.93, 92.75], eta partial squared= .874  Anterior-posterior p< .001  F=108, gl= [1.16, 55.83], eta partial squared= .692  Laterality x Anterior-posterior p< .001  F= 66.22, gl= [2.47, 118.41], eta partial squared= .580  **Laterality x Anterior-posterior x group p= .043***  F= 3, gl= [2.47, 118.41], eta partial squared= .059 |
| **BETA**  **(13-20 Hz)** | **Within-subjects:**  Laterality p< .001  F=51.08, gl= [1.64, 101.56], eta partial squared= .452  Anterior-posterior p= .002  F=7.36, gl= [1.58, 97.92], eta partial squared= .106  Laterality x Anterior-posterior p< .001  F=28.76, gl= [3.66, 226.65], eta partial squared= .317 | **Within-subjects:**  Laterality p< .001  F=77.06, gl= [1.47, 70.96], eta partial squared= .616  Anterior-posterior p< .001  F=35.85, gl= [1.63, 78.28], eta partial squared= .428  Laterality x Anterior-posterior p< .001  F=24.91, gl= [3.09, 148.28], eta partial squared= .342 |

| **Supplementary Table 6.** | |
| --- | --- |
| Significant results of the ANOVA analysis of the mean of absolute PSD values, with factors group of subjects (control and ADHD), laterality, anterior-posterior and open eyes and closed eyes conditions (OE & CE), for each band independently. All significant results are displayed. The results in which the factor group was significant as a main or interactive effect are indicated in bold. | |
| **DELTA**  **(1-2 Hz)** | **Within-subjects:**  OE-CE p < .001*****  F =59.78, gl = [1, 46], eta partial squared = .565  **OE-CE x group p= .025***  F =5.4, gl = [1, 46], eta partial squared = .105  Laterality p < .001*  F = 135.8, gl = [1.91, 87.91], eta partial squared = .747  Anterior-posterior p < .001*****  F = 328.92, gl = [1.24, 57.03], eta partial squared = .877  OE-CE x Laterality p < .001*****  F = 12.6, gl = [1.92, 88.12], eta partial squared = .215  OE-CE x Anterior-posterior p < .001*****  F = 50.14, gl = [1.34, 61.79], eta partial squared = .522  Laterality x Anterior-posterior p < .001*****  F = 54.24, gl = [3.33, 153.31], eta partial squared = .541 |
| **THETA**  **(4-7 Hz)** | **Within-subjects:**  OE-CE p < .001*****  F =53.02, gl = [1, 46], eta partial squared = .535  Laterality p < .001*  F = 657.3, gl = [1.95, 89.84], eta partial squared = .935  Anterior-posterior p < .001*****  F = 98.81, gl = [1.39, 64.07], eta partial squared = .682  OE-CE x Laterality p= .001*****  F = 7.92, gl = [1.98, 91.09], eta partial squared = .147  OE-CE x Anterior-posterior p < .001*****  F = 22.7, gl = [1.41, 64.77], eta partial squared = .330  Laterality x Anterior-posterior p < .001*****  F = 149.73, gl = [3.22, 147.98], eta partial squared = .765 |
| **ALPHA**  **(8-11 Hz)** | **Within-subjects:**  OE-CE p < .001*****  F =144.31, gl = [1, 46], eta partial squared = .758  Laterality p < .001*  F = 415.39, gl = [1.92, 88.49], eta partial squared = .900  Anterior-posterior p < .001*****  F = 56.31, gl = [1.18, 54.3], eta partial squared = .550  OE-CE x Anterior-posterior p < .001*  F = 101.62, gl = [1.43, 66.03], eta partial squared = .688  Laterality x Anterior-posterior p < .001*****  F = 84.51, gl = [2.53, 116.58], eta partial squared = .648  OE-CE x Laterality x Anterior-posterior p= .028*  F = 3.22, gl = [2.8, 128.31], eta partial squared = .065 |
| **BETA**  **(13-20 Hz)** | **Within-subjects:**  OE-CE p < .001*****  F =45.29, gl = [1, 46], eta partial squared = .496  Laterality p < .001*  F = 64.03, gl = [1.5, 69.24], eta partial squared = .582  Anterior-posterior p < .001*****  F = 10.12, gl = [1.61, 74.06], eta partial squared = .180  OE-CE x Laterality p < .001*  F = 9.83, gl = [1.94, 89.28], eta partial squared = .176  OE-CE x Anterior-posterior p < .001*  F = 77.34, gl = [1.58, 72.69], eta partial squared = .627  Laterality x Anterior-posterior p < .001*****  F = 24.79, gl = [3.28, 150.78], eta partial squared = .350 |

| **Supplementary Table 7** | | | | |
| --- | --- | --- | --- | --- |
| Spearman correlations of the mean of absolute PSD in the different analyzed frequencies vs. age (expressed in days) for control and ADHD group in Open Eyes condition (OE). *P-values* are corrected for multiple comparisons with FDR. The PSD values of all electrodes are collapsed. Notice that all significant correlations are negative. | | | | |
| **Frequencies** | **CONTROL** | | **ADHD** | |
|  | **R** | ***P*** | **R** | ***p*** |
| 1 | -.743 | < .001 | -.765 | < .001 |
| 2 | -.809 | < .001 | -.826 | < .001 |
| 3 | -.831 | < .001 | -.827 | < .001 |
| 4 | -.786 | < .001 | -.820 | < .001 |
| 5 | -.766 | < .001 | -.820 | < .001 |
| 6 | -.806 | < .001 | -.816 | < .001 |
| 7 | -.773 | < .001 | -.761 | < .001 |
| 8 | -.795 | < .001 | -.748 | < .001 |
| 9 | -.772 | < .001 | -.710 | < .001 |
| 10 | -.605 | < .001 | -.646 | < .001 |
| 11 | -.346 | .062 | -.330 | .072 |
| 12 | -.143 | .456 | -.182 | .319 |
| 13 | -.088 | .630 | -.234 | .208 |
| 14 | -.218 | .256 | -.346 | .061 |
| 15 | -.424 | .019 | -.350 | .062 |
| 16 | -.487 | .006 | -.387 | .037 |
| 17 | -.549 | .002 | -.451 | .013 |
| 18 | -.581 | < .001 | -.474 | .009 |
| 19 | -.594 | < .001 | -.512 | .005 |
| 20 | -.523 | .003 | -.537 | .003 |

| **Supplementary Table 8.** | | | | |
| --- | --- | --- | --- | --- |
| Spearman correlations of the mean of absolute PSD in the different analyzed frequencies vs. age (expressed in days) for the control and ADHD groups in the Closed Eyes condition (CE). *P-values* are corrected for multiple comparisons with FDR. The PSD values of all electrodes are collapsed. Notice that all significant correlations are negative. | | | | |
| **Frequencies** | **CONTROL** | | **ADHD** | |
|  | **R** | ***P*** | **R** | ***p*** |
| 1 | -.701 | < .001 | -.618 | .002 |
| 2 | -.684 | < .001 | -.665 | < .001 |
| 3 | -.737 | < .001 | -.668 | .001 |
| 4 | -.800 | <.001 | -.683 | < .001 |
| 5 | -.817 | < .001 | -.722 | < .001 |
| 6 | -.755 | <.001 | -.699 | < .001 |
| 7 | -.715 | <.001 | -.707 | < .001 |
| 8 | -.656 | < .001 | -.610 | .003 |
| 9 | -.652 | < .001 | -.428 | .073 |
| 10 | -.359 | .104 | -.130 | .595 |
| 11 | .057 | .828 | .011 | .959 |
| 12 | .278 | .222 | -.012 | 1 |
| 13 | .180 | .432 | -.151 | .629 |
| 14 | -.025 | .904 | -.148 | .598 |
| 15 | -.212 | .365 | -.215 | .466 |
| 16 | -.398 | .069 | -.238 | .418 |
| 17 | -.447 | .042 | -.300 | .290 |
| 18 | -.455 | .041 | -.291 | .288 |
| 19 | -.471 | .035 | -.195 | .498 |
| 20 | -.399 | .074 | -.138 | .599 |

| **Table Supplementary 9.** | | | | |
| --- | --- | --- | --- | --- |
| Standard Deviation of PSD (SDp) vs. age (expressed in days) Spearman correlations (Rho) for control and ADHD group in open eyes condition (OE). The standard deviations of all electrodes are collapsed. *P-values* with FDR corrections multiple comparisons. | | | | |
| **Frequencies** | **CONTROL** | | **ADHD** | |
|  | **Rho** | ***p*** | **Rho** | ***p*** |
| 1 | -.754 | < .001 | -.773 | < .001 |
| 2 | -.804 | < .001 | -.826 | < .001 |
| 3 | -.829 | < .001 | -.826 | < .001 |
| 4 | -.783 | < .001 | -.813 | < .001 |
| 5 | -.765 | < .001 | -.813 | < .001 |
| 6 | -.810 | < .001 | -.813 | < .001 |
| 7 | -.772 | < .001 | -.755 | < .001 |
| 8 | -.786 | < .001 | -.736 | < .001 |
| 9 | -.760 | < .001 | -.701 | < .001 |
| 10 | -.589 | < .001 | -.643 | < .001 |
| 11 | -.320 | .088 | -.299 | .107 |
| 12 | -.135 | .487 | -.139 | .449 |
| 13 | -.095 | .605 | -.214 | .251 |
| 14 | -.221 | .249 | -.327 | .079 |
| 15 | -.412 | .023 | -.350 | .061 |
| 16 | -.484 | .006 | -.384 | .039 |
| 17 | -.554 | .002 | -.461 | .011 |
| 18 | -.584 | < .001 | -.480 | .008 |
| 19 | -.596 | < .001 | -.509 | .004 |
| 20 | -.521 | .003 | -.547 | .002 |

| **Table Supplementary 10.** | | | | |
| --- | --- | --- | --- | --- |
| Standard Deviation of PSD (SDp) vs. age (expressed in days) Spearman correlations (Rho) for control and ADHD group in closed eyes condition (CE). The standard deviation of all electrodes is collapsed. *P-values* with FDR corrections multiple comparisons. | | | | |
| **Frequencies** | **CONTROL** | | **ADHD** | |
|  | **Rho** | ***p*** | **Rho** | ***p*** |
| 1 | -.718 | < .001 | -.566 | .007 |
| 2 | -.666 | < .001 | -.651 | .001 |
| 3 | -.735 | < .001 | -.657 | .001 |
| 4 | -.788 | < .001 | -.683 | < .001 |
| 5 | -.817 | < .001 | -.722 | < .001 |
| 6 | -.757 | < .001 | -.685 | .001 |
| 7 | -.717 | < .001 | -.695 | .001 |
| 8 | -.635 | .001 | -.616 | .002 |
| 9 | -.634 | .001 | -.417 | .084 |
| 10 | -.355 | .109 | -.125 | .650 |
| 11 | .066 | .793 | -.018 | .979 |
| 12 | .288 | .203 | -.004 | .985 |
| 13 | .182 | .426 | -.112 | .661 |
| 14 | .010 | .962 | -.166 | .569 |
| 15 | -.198 | .401 | -.225 | .431 |
| 16 | -.398 | .069 | -.232 | .442 |
| 17 | -.428 | .054 | -.288 | .323 |
| 18 | -.460 | .038 | -.276 | .329 |
| 19 | -.496 | .035 | -.175 | .573 |
| 20 | -.405 | .068 | -.126 | .684 |

| **Supplementary Table 11.** | | |
| --- | --- | --- |
| Significant results obtained in the ANOVA analysis of the coefficient of variation values (CV) with factors: group of subjects, anterior-posterior, and laterality. The ANOVA was computed independently for the open (OE) and closed eyes conditions (CE). The results in which the factor group was significant as a main or interactive effect are indicated in bold. | | |
| **BANDS** | **OA** | **OC** |
| **DELTA**  **(1-2 Hz)** | **Between-subjects:**  **Group p= .041***  F= 4.37, gl= [1, 62], eta partial squared= .066  **Within-subjects:**  Laterality p= .017  F=4.37, gl= [1.84, 114.09], eta partial squared= .066  Anterior-posterior p< .001  F= 12.47, gl= [1.69, 105.02], eta partial squared= .167 | **Between-subjects:**  **Group p= .002***  F= 10.21, gl= [1, 48], eta partial squared= .175  **Within-subjects:**  Anterior-posterior p= .038  F= 3.68, gl= [1.65, 79.11], eta partial squared= .071 |
| **THETA**  **(4-7 Hz)** | **Within-subjects:**  Laterality p= .010  F=5.24, gl= [1.69, 104.8], eta partial squared= .078  Anterior-posterior p< .001  F=44.01, gl= [1.15, 71.37], eta partial squared= .415  **Anterior-posterior x group p= .019***  F= 5.36, gl= [1.15, 71.37], eta partial squared= .08  Laterality x Anterior-posterior p= .028  F= 3.22, gl= [2.72, 168.42], eta partial squared= .049 | **Within-subjects:**  Anterior-posterior p< .001  F=23.72, gl= [1.67, 80.22], eta partial squared= .331 |
| **ALPHA**  **(8-11 Hz)** | **Within-subjects:**  Anterior-posterior p< .001  F= 32.19, gl= [1.35, 83.63], eta partial squared= .342 | **Within-subjects:**  Laterality p= .025  F=3.97, gl= [1.86, 89.18], eta partial squared= .076  Laterality x Anterior-posterior p< .034  F= 2.74, gl= [3.66, 175.47], eta partial squared= .054 |
| **BETA**  **(13-20 Hz)** | **Within-subjects:**  **Anterior-posterior x group p= .004***  F=6.95, gl= [1.53, 94.75], eta partial squared= .101  Laterality x Anterior-posterior p= .004  F=4.32, gl= [3.43, 212.63], eta partial squared= .065 |  |

| **Supplementary Table 12.** | |
| --- | --- |
| Significant results of the ANOVA analysis of the coefficients of variation values (CV), with factors group of subjects (control and ADHD), laterality, anterior-posterior and open eyes and closed eyes conditions (OE & CE), for each band independently. All significant results are displayed. The results in which the factor group was significant as a main or interactive effect are indicated in bold. | |
| **DELTA**  **(1-2 Hz)** | **Between-subjects:**  **group p= .002***  F = 10.97, gl = [1, 46], eta partial squared = .193  **Within-subjects:**  Anterior-posterior p < .001  F = 12.27, gl = [2, 76.88], eta partial squared = .211 |
| **THETA**  **(4-7 Hz)** | **Within-subjects:**  **OE-CE x group p= .009***  F =7.41, gl = [1, 46], eta partial squared = .139  Laterality p= .012  F = 5.33, gl = [1.57, 72.13], eta partial squared = .104  Anterior-posterior p < .001  F = 48.61, gl = [1.27, 58.4], eta partial squared = .514  **Anterior-posterior x group p= .043***  F = 3.92, gl = [1.27, 58.4], eta partial squared = .078  OE-CE x Anterior-posterior p= .013  F = 5.34, gl = [1.47, 67.4], eta partial squared = .104  **OE-CE x Anterior-posterior x group p= .044***  F = 3.65, gl = [1.47, 67.4], eta partial squared = .074 |
| **ALPHA**  **(8-11 Hz)** | **Within-subjects:**  OE-CE p < .001  F =28.5, gl = [1, 46], eta partial squared = .383  Laterality p= .007  F = 5.82, gl = [1.63, 74.96], eta partial squared = .112  Anterior-posterior p < .001  F = 21.47, gl = [1.4, 64.38], eta partial squared = .318  OE-CE x Anterior-posterior p < .001  F = 17.41, gl = [1.24, 56.9], eta partial squared = .275  Laterality x Anterior-posterior p=.030  F = 3.27, gl = [2.57, 118.24], eta partial squared = .066  OE-CE x Laterality x Anterior-posterior p= .039  F = 3.18, gl = [2.29, 105.24], eta partial squared = .065 |
| **BETA**  **(13-20 Hz)** | **Within-subjects:**  **Anterior-posterior x group p= .023***  F = 4.76, gl = [1.36, 62.74], eta partial squared = .094  **OE-CE x Anterior-posterior x group p= .05***  F = 3.5, gl = [1.46, 67.16], eta partial squared = .071  OE-CE x Laterality x Anterior-posterior p= .025  F = 3.28, gl = [2.88, 132.56], eta partial squared = .067 |
